# Transcriptomic analysis of glutamate-induced HT22 neurotoxicity as a model for screening anti-Alzheimer’s drugs

**DOI:** 10.1038/s41598-023-34183-y

**Published:** 2023-05-04

**Authors:** Anchalee Prasansuklab, Suporn Sukjamnong, Atsadang Theerasri, Valerie W. Hu, Tewarit Sarachana, Tewin Tencomnao

**Affiliations:** 1 Natural Products for Neuroprotection and Anti-ageing Research Unit, Chulalongkorn University, Bangkok, 10330 Thailand; 2 College of Public Health Sciences, Chulalongkorn University, Bangkok, 10330 Thailand; 3 Department of Clinical Chemistry, Faculty of Allied Health Sciences, Chulalongkorn University, Bangkok, 10330 Thailand; 4 SYstems Neuroscience of Autism and PSychiatric Disorders (SYNAPS) Research Unit, Department of Clinical Chemistry, Faculty of Allied Health Sciences, Chulalongkorn University, Bangkok, Thailand; 5 Department of Biochemistry and Molecular Medicine, The George Washington University School of Medicine and Health Sciences, The George Washington University, Washington, DC USA

**Keywords:** Biochemistry, Molecular biology, Neurodegenerative diseases, Drug discovery and development

## Abstract

Glutamate-induced neurotoxicity in the HT22 mouse hippocampal neuronal cell line has been recognized as a valuable cell model for the study of neurotoxicity associated with neurodegenerative diseases including Alzheimer’s disease (AD). However, the relevance of this cell model for AD pathogenesis and preclinical drug screening remains to be more elucidated. While there is increasing use of this cell model in a number of studies, relatively little is known about its underlying molecular signatures in relation to AD. Here, our RNA sequencing study provides the first transcriptomic and network analyses of HT22 cells following glutamate exposure. Several differentially expressed genes (DEGs) and their relationships specific to AD were identified. Additionally, the usefulness of this cell model as a drug screening system was assessed by determining the expression of those AD-associated DEGs in response to two medicinal plant extracts, *Acanthus ebracteatus* and *Streblus asper*, that have been previously shown to be protective in this cell model. In summary, the present study reports newly identified AD-specific molecular signatures in glutamate-injured HT22 cells, suggesting that this cell can be a valuable model system for the screening and evaluation of new anti-AD agents, particularly from natural products.

## Introduction

Alzheimer’s disease (AD) is the most common type of neurodegenerative disorders (NDDs) and the most common cause of dementia in the elderly, accounting for at least two-thirds of patients^1^. AD is characterized by the progressive loss of the structure and function of neuronal cells, accompanied by aberrant accumulation of β-amyloid plaques and tau-containing neurofibrillary tangles in the brain which are considered unique hallmarks of AD^2^. While no cure for AD currently exists, the number of patients with this disease globally is rising rapidly each year^3^. Enormous efforts have been spent on drug discovery in the past decades, but only few compounds have been approved by Food and Drug Administration (FDA) for relieving AD symptoms. So far, cholinesterase inhibitors (donepezil, rivastigmine, galantamine) and an antagonist of the *N*-methyl-d-aspartate receptor (memantine) have shown to temporarily improve cognitive functions such as attention, memory, and learning, but all without halting or reversing disease progression^4^. The continued failure of AD drug development has emphasized an urgent need for novel therapy in the near future.

Considering that AD is multifactorial and involved with several pathogenic mechanisms, a paradigm shift in therapeutic strategy from a conventional target acetylcholinesterase (AChE) is clearly warranted for effective AD drug development^5^. Glutamate is one of the most abundant amino acids in the human body and plays a crucial role in the central nervous system (CNS) as a principal excitatory neurotransmitter for a variety of normal brain functions. However, glutamate concentration must be maintained at an optimal level within the extracellular space. An abnormal increase in the extracellular glutamate level in the brain can cause toxicity that eventually leads to neuronal cell death^6^. There has been consistent evidence that glutamate toxicity drives neurodegeneration in several types of NDDs including AD^7^. Therefore, the biological pathways underlying glutamate neurotoxicity are thus of great therapeutic interest and serve as another effective target in counteracting AD progression^8^.

One of the key factors for successful AD drug development is utilizing a suitable in vitro disease model in preclinical drug screening^9^. In the pursuit of medicines for NDDs therapy over the past few decades, various neuronal cell lines have been employed as a model of glutamate-mediated neurodegeneration for investigating potential natural compounds with anti-glutamate toxicity^10^. However, it should be noted that different cell lines may differ considerably in their responses when exposed to glutamate. Those cell lines include PC12 (rat adrenal medulla pheochromocytoma), SH-SY5Y (human neuroblastoma), C6 (rat glioma cells), RGC-5 (retinal ganglion cells), Neuro2a (mouse neuroblastoma), and HT22 (mouse hippocampal cells) which is the most popular among all models^10,11^.

The HT22 mouse hippocampal neuronal cell line is currently recognized as a valuable experimental model for the study of glutamate-induced neurotoxicity associated with NDDs^6^. Nevertheless, the relevance of this cell model for AD pathogenesis and preclinical drug screening remains to be elucidated. While there is an increasing use of this cell model in a number of studies, still little is known about its underlying molecular signatures in relation to AD. To date, a limited number of genes and signaling proteins were analyzed in previous studies of glutamate-induced HT22 cells, with a major focus on oxidative stress, ER stress, mitochondrial dysfunction and apoptotic cell death^6,12,13^.

With the use of this cell model, several plants and natural products have been extensively studied for their neuroprotective potential, including the medicinal plants *Acanthus ebracteatus* (AE) and *Streblus asper* (SA) reported by our research group^14,15^. We have demonstrated that ethanolic extracts of both AE and SA leaves prepared using Soxhlet extraction technique, could protect against HT22 cell death from glutamate-induced toxicity via inhibition of mitochondrial apoptotic-inducing factor (AIF)-dependent apoptosis. Co-treatment of these extracts could also suppress the production of intracellular reactive oxygen species (ROS) following high glutamate exposure through activation of the main antioxidant system, nuclear factor erythroid 2-related factor 2 (Nrf2) pathway, leading to upregulated expressions of antioxidant-related Nrf2 downstream genes including NAD(P)H quinone dehydrogenase 1 (*NQO1*), glutamate-cysteine ligase modifier subunit (*GCLM*), and solute carrier family 1 member 1 (*SLC1A1*/EAAT3). Nevertheless, further investigations regarding the usefulness and application of both plant extracts in AD are still warranted.

Due to recent advances in next generation sequencing, transcriptomic analyses combined with bioinformatic tools are considered a powerful approach to provide a more comprehensive view of the gene expression levels for entire genomes, thereby allowing unbiased identification of both known and novel target genes for elucidating the mechanisms of action under conditions of interest. Thus, in the present study, we attempted to clarify the genome-wide gene expression changes in HT22 cells following glutamate exposure using transcriptome sequencing (RNA-Seq). The differentially expressed genes (DEGs) were subsequently analyzed for genes and pathways that could be linked to AD as well as the disease-specific molecular signatures that could reveal new druggable targets. Additionally, we assessed the usefulness of this cell model as a drug screening system by determining the expression level of the AD-associated DEGs in response to the extracts of two medicinal plants, AE and SA, which have shown protective activities in previous reports from our group^14,15^.

## Materials and methods

### Cell culture

A mouse hippocampal HT22 cell line, kindly provided by Professor David Schubert at the Salk Institute, San Diego, CA, USA, was maintained in Dulbecco's modified Eagle medium (DMEM) containing 10% (v/v) fetal bovine serum (FBS), 100 U/mL penicillin and 100 μg/mL streptomycin. The cells were grown in a humidified incubator (5% CO_2_, 37 °C) and passaged when reaching 70% confluency. The cells were seeded and incubated overnight prior to treatment.

### Treatment conditions and RNA isolation

HT22 cells were treated with only 5 mM glutamate or glutamate in combination with either AE or SA extract for 24 h under conditions previously reported to be neuroprotective^14,15^ (Supplementary Fig. S1). After the treatment, total RNA was extracted using Trizol reagent (Invitrogen, USA) following the manufacturer’s procedure. RNA quality and quantity were analyzed using a Nanodrop™ 2000 spectrophotometer (Thermo Fisher Scientific, USA). The absorbance ratio of A260/A230 between 2.0 and 2.2 and ratio of A260/A280 around 2.0 are accepted as pure for further analysis.

### Transcriptome sequencing (RNA-Seq) and analysis

To identify differentially expressed genes (DEGs) in response to glutamate exposure, transcriptome profiling was performed by BGI Genomics Co., Ltd. using the Illumina HiSeq™ 2000 next-generation sequencing platform (Illumina, San Diego, CA, USA) according to the manufacturer’s protocol. Briefly, total RNA was treated with DNase I, and the oligo(dT) treatment was used for mRNA isolation. Next, the RNA was mixed with fragmentation buffer to fragment the mRNA. Then, cDNA was synthesized using the mRNA fragments as templates. The double stranded cDNA was purified with magnetic beads. End reparation and 3'end single nucleotide A (adenine) addition were then performed. After that, sequencing adaptors were ligated to the fragments, and suitable fragments were selected for PCR amplification. An Agilent 2100 Bioanalyzer (Agilent Technologies, Santa Clara, CA, USA) and an ABI StepOnePlus Real-Time PCR System (Applied Biosystems, Waltham, MA, USA) were used for quantification and quality control of the sample library. The library was then sequenced using Illumina HiSeq™ 2000 (Illumina, San Diego, CA, USA). Subsequently, sequencing reads were filtered and subjected to quality control. Clean reads in a FASTQ file were mapped to the reference genome using Bowtie, and gene expression levels were then calculated using RSEM. The transcriptome profiles between treatment groups were compared using a Poisson distribution. P-values were calculated using a Poisson distribution method. DEGs with a P-value < 0.05 and FDR < 0.05 were considered statistically significant.

### Prediction of biological functions and interactome analysis

Biological functions, disorders, canonical pathways, and interactome networks associated with DEGs were predicted using Ingenuity Pathway Analysis (IPA) software (Qiagen, Inc., USA). The Fisher’s exact test was used to calculate P-values for sets of DEGs enriched in experimentally validated genes for known biological functions, disorders, and canonical pathways annotated in the Ingenuity Knowledge Base. A P-value < 0.05 was considered statistically significant.

### Quantitative reverse transcription PCR (RT-qPCR)

One μg of total RNA was used for cDNA synthesis using AccuPower® RT PreMix (Bioneer, South Korea) and oligo (dT) 17-mer. The condition for reverse transcription was started by initial incubation at 70 °C for 5 min, followed by 42 °C for 60 min and 94 °C for 5 min. Then, the reverse-transcribed products were used as cDNA templates for amplification which was performed using AccuPower® 2 × Greenstar™ qPCR Mastermix (Bioneer). Specific primers for *Apoe, Ptgs2, Rest, Zbed6, Loxl2, Ccl2, Synpo, Ablim1, Glis3*, and *β-actin* are provided in Supplementary Table S7. The reverse transcription and Real-time PCR reactions were carried out using Exicycler™ 96 (Bioneer). The thermal cycling conditions were set as follows: an initial denaturing step at 95 °C for 10 min, 45 cycles of 95 °C for 15 s, 58 °C for 15 s and 72 °C for 30 s. Melting curve analysis was examined at 65–95 °C to verify the amplicons. Gene expression levels were calculated by 2^−ΔΔCT^ method using *β-actin* as an internal control.

### Statistical analysis

The results are presented as the mean ± SEM. The results of 3 repeats were analyzed using one-way ANOVA with post hoc Dunnett’s tests (Prism 6, Graph-Pad, CA, USA). The differences were considered statistically significant at a P-value < 0.05.

## Results

### The quality of RNA sequencing data

After filtering the low-quality reads, high-quality clean reads of 5mMGlu, vehicle control, AE-Et50, and SA-Et50 sample were 12,164,574 (99.77% of the raw reads), 12,161,689 (99.75% of the raw reads), 12,160,701 (99.74% of the raw reads), and 12,159,420 (99.73% of the raw reads), respectively. Clean reads were mapped to reference sequences. Greater than 86.74% of the total clean reads from the RNA-Seq data were aligned and mapped uniquely to the reference genome for all samples.

### Expression profiling of DEGs among treatment conditions in cultured HT22 cells

**Figure 1:**
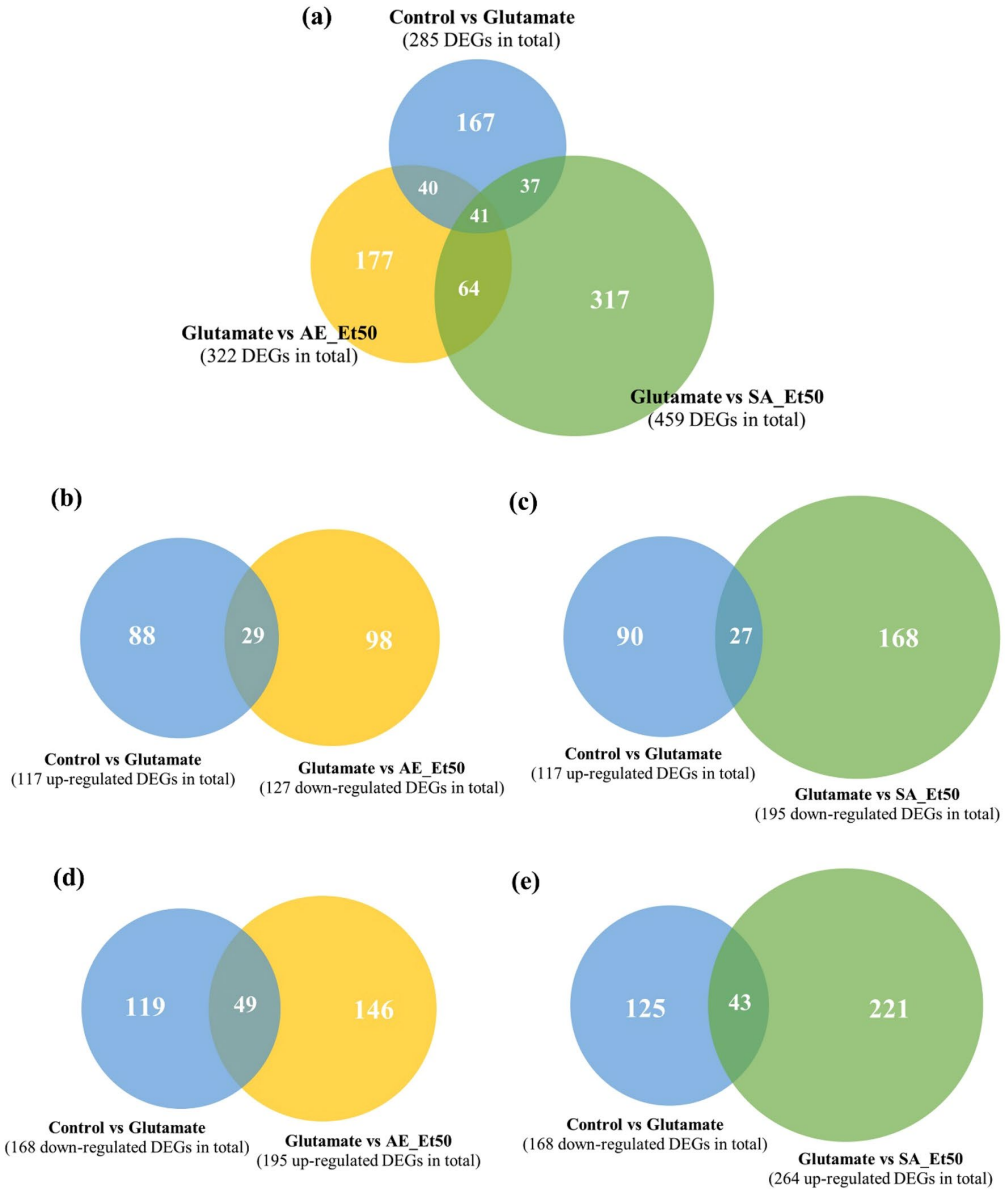
Venn diagram of the overlapping DEGs between treatment groups. (**a**) An overall comparison showing 41 genes common to the three pairwise treatment conditions. (**b**) The intersection between up-regulated DEGs induced by glutamate treatment and down-regulated DEGs following glutamate plus AE extract or (**c**) SA extract. (**d**) The intersection between down-regulated DEGs induced by glutamate treatment and up-regulated DEGs following glutamate plus AE extract or (**e**) SA extract.

**Figure 2:**
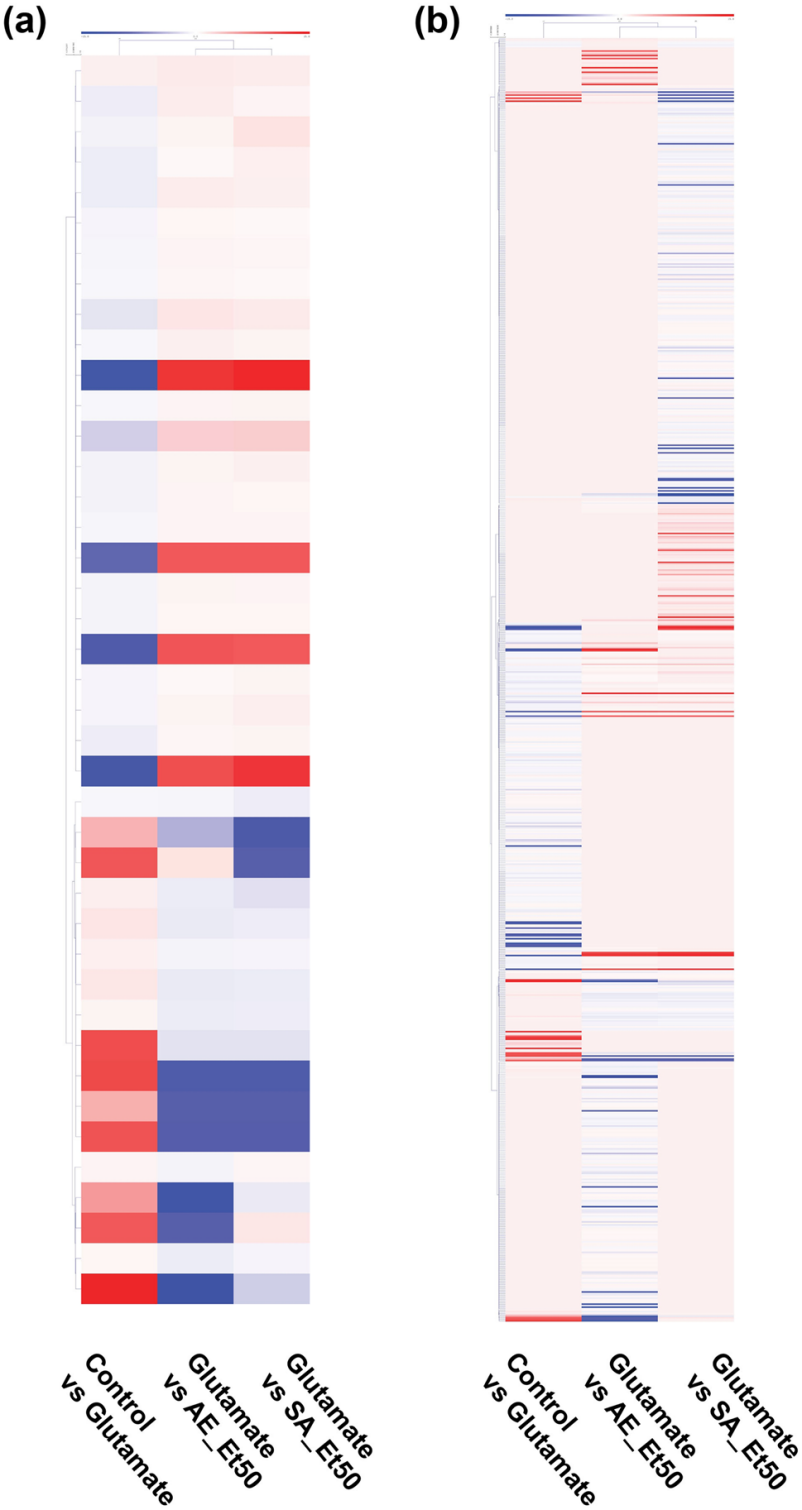
Hierarchical clustering heatmap of DEGs among treatment conditions in HT22 cells. (**a**) Heatmap analyses of DEGs obtained using the hierarchical clustering method based on the expression pattern of the overlapping gene set (41 DEGs) from a three-way comparison of DEGs from the different experimental groups, and (**b**) the complete gene sets (285/322/459 DEGs) resulting from the three treatment-comparison groups. Each row represents a gene, and each column represents each comparison group as indicated at the bottom. The color bar represents the log2 fold change in expression level and ranges from blue (downregulation) to red (upregulation).

To investigate whether glutamate treatment ± AE or SA extracts alter the transcriptome profiles of cultured HT22 cells, we conducted separate RNA-seq analyses of HT22 cells treated with glutamate, co-treated with glutamate and AE extract, and co-treated with glutamate and SA extract. We found that 285 genes were significantly differentially expressed with glutamate treatment compared with the vehicle control, while 322 and 459 genes were significantly differentially expressed in cells that were co-treated with glutamate + AE extract and glutamate + SA extract, respectively (*P* < 0.05 and FDR < 0.05; Table 1) relative to the glutamate-treated cells. The lists of DEGs are shown in Supplementary Tables S1–S3. The results of Venn diagram analyses representing overlapping DEGs are shown in Fig. 1 and Supplementary Table S4. The heatmaps of DEGs are shown in Fig. 2.

**Table 1:** Summary of DEGs between treatment groups in the study.

Comparisons	Up-regulated genes	Down-regulated genes	Total DEGs
Control vs Glutamate	117 (41.1%)	168 (58.9%)	285
Glutamate vs AE_Et50	195 (60.6%)	127 (39.4%)	322
Glutamate vs SA_Et50	264 (57.5%)	195 (42.5%)	459

### Biological pathways and network analyses of DEGs among treatment conditions in cultured HT22 cells

**Figure 3:**
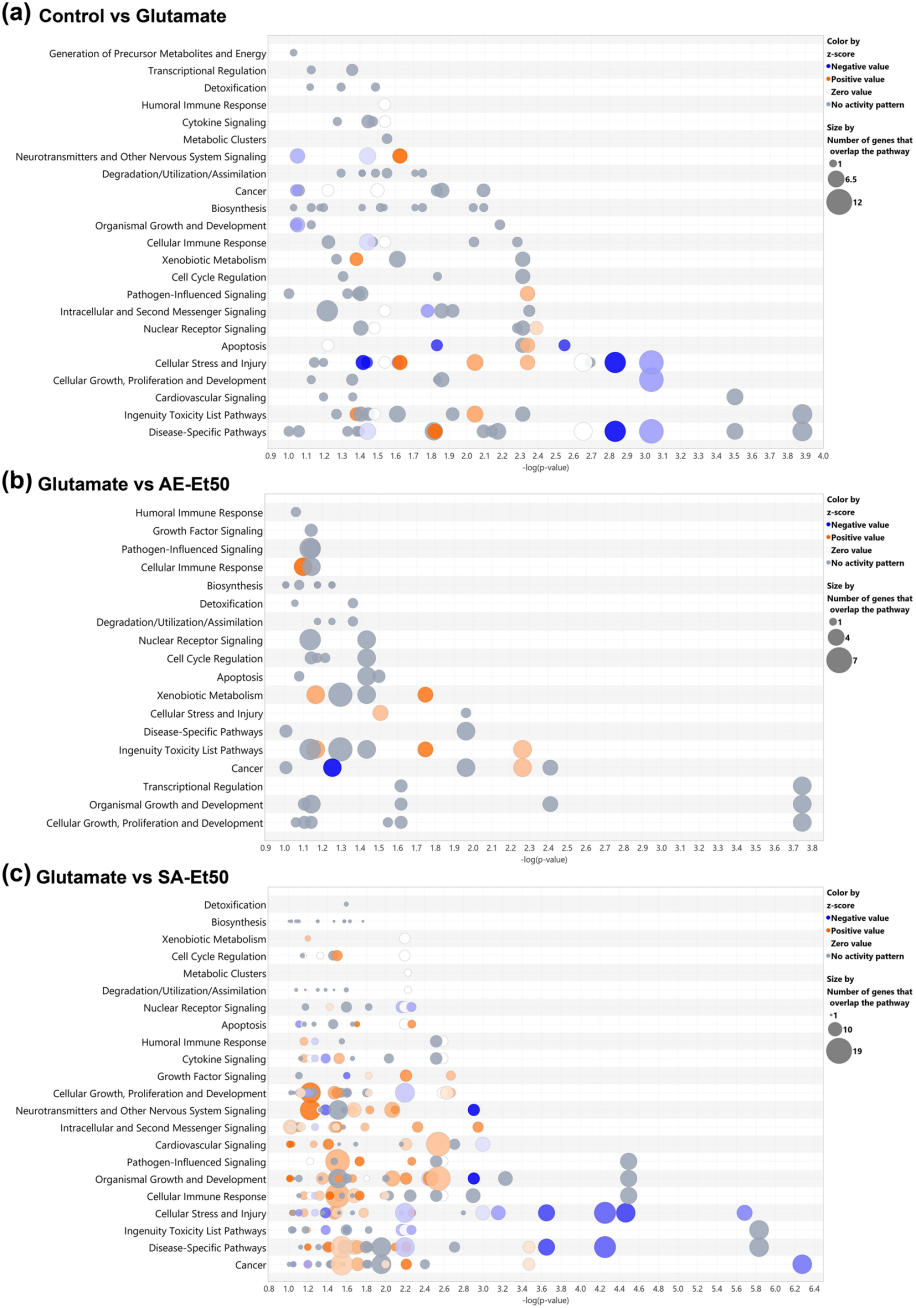
The canonical pathways associated with DEGs identified from cells treated with glutamate (**a**), glutamate with AE extract (**b**), and glutamate with SA extract (**c**). The list of DEGs from RNA-seq analysis were analyzed using IPA software to predict canonical pathways associated with DEGs. Bubble plot indicates the canonical pathways, where bubble size corresponds to number of genes enriched for corresponding pathway and color indicates z-score. Orange bubbles indicate predicted activation and a positive z-score, blue bubbles indicate predicted inhibition and a negative z-score.

The lists of DEGs among treatment conditions in HT22 cells were analyzed using IPA software to predict canonical pathways, diseases/disorders, and biological functions significantly associated with DEGs. The bubble chart of canonical pathway analysis (Fig. 3a) showed that DEGs induced by glutamate treatment were significantly associated with the changes of canonical pathways in “Neurotransmitters and Other Nervous System Signaling” category, including the activation state of Neurovascular Coupling Signaling Pathway (DEGs: CACNA1C, ENTPD5,GRM1, PLA2G4A, PLA2G4B, and PTGS2) and the inhibition state of Neuroinflammation Signaling Pathway (DEGs: HMOX1, IKBKG, IL1R1, PLA2G4A, PLA2G4B, PTGS2, and TLR1) and Synaptogenesis Signaling Pathway (DEGs: APOE, GRM1, LRP1, NLGN2, THBS1, and THBS2).

We found that DEGs from cells co-treated with AE extracts were significantly associated with the changes of canonical pathways in “Cellular Immune Response” category (Fig. 3b), including the activation state of Natural Killer Cell Signaling Pathway (DEGs: CD247, HLA-F, MAP3K1, NFAT5, and ULBP1) and “Ingenuity Toxicity List Pathways” category, including the activation state of p53 Signaling Pathway (DEGs: APAF1, CCNG1, HIPK2, RB1, and THBS1), Xenobiotic Metabolism AHR Signaling Pathway (DEGs: ALDH3A1, HDAC5, NRIP1, and UGT1A6), and Xenobiotic Metabolism CAR Signaling Pathway (DEGs: ALDH3A1, NRIP1, PPM1L, SOD3, and UGT1A6).

In addition, our results revealed that DEGs from cells co-treated with SA extracts were significantly associated with the changes of canonical pathways in “Neurotransmitters and Other Nervous System Signaling” category (Fig. 3c), including the activation state of CREB Signaling in Neurons Pathway (DEGs: ACKR3, CAMK2B, CMKLR1, GPR162, GPRC5A, GPRC5B, GRIK2, GRM1, P2RY14, PDGFB, PIK3R1, PIK3R5, PLCD3, PRKAG2, S1PR1, and TGFB1), Neuropathic Pain Signaling In Dorsal Horn Neurons Pathway (DEGs: CAMK2B, GRM1, PIK3R1, PIK3R5, PLCD3, and PRKAG2), Amyotrophic Lateral Sclerosis Signaling (DEGs: APAF1, CAPN5, GLUL, GRIK2, PIK3R1, and PIK3R5), Reelin Signaling in Neurons (DEGs: APOE, AMK2B, ITGA5, PDK2, PIK3R1, and PIK3R5), Synaptogenesis Signaling Pathway (DEGs: APOE, CAMK2B, GRM1, ITSN2, NECTIN1, NRXN2, PIK3R1, PIK3R5, PRKAG2, SYT7, THBS1, and THBS2), Neuroinflammation Signaling Pathway (DEGs: GLUL, HLA-F, HMOX1, NFAT5, PIK3R1, PIK3R5, PLA2G4B, PTGS2, SLC1A3, TGFB1, and TLR1) and the inhibition state of Neurovascular Coupling Signaling Pathway (DEGs: ENTPD6, GRM1, KCNJ15, NPR1, PLA2G4B, PRKAG2, PTGS2, and SLC1A3) and Semaphorin Neuronal Repulsive Signaling Pathway (DEGs: CD44, CFL2, CSPG4, ITGA5, ITGB2, PIK3R1, PIK3R5, PRKAG2, and VCAN).

We found that “neurological disease” was present among the top diseases/disorders significantly associated with DEGs from cells treated with glutamate relative to vehicle control and DEGs from cells co-treated with glutamate and AE extracts relative to the glutamate treated cells (*P* < 0.05; Supplementary Table S5). DEGs from cells co-treated with glutamate and SA extracts relative to the glutamate treated cells also were significantly associated with “neurological disease” (P = 1.49E−06 to 4.47E−15). Our results revealed that DEGs induced by glutamate treatment were significantly associated with neurological disorders and diseases, including “cerebral disorder”, central nervous system cancer, “brain glioma”, “glioma”, and “cerebrovascular dysfunction” (*P* < 0.05; Supplementary Table S6). In addition, several nervous system functions, including “sensory system development”, “activation of microglia”, “differentiation of synapse”, “morphogenesis of nervous tissue”, and “neuritogenesis” were also significantly associated with DEGs (*P* < 0.05; Supplementary Table S6). Furthermore, we found that DEGs from cells co-treated with glutamate and AE extracts were associated with several neurological diseases, including “early-onset Alzheimer’s disease” (*P* < 0.05; Supplementary Table S6). Our results revealed that both sets of DEGs from cells co-treated with AE extracts and cells co-treated with SA extracts were significantly associated with “development of neurons” (*P* < 0.05; Supplementary Table S6).

**Figure 4:**
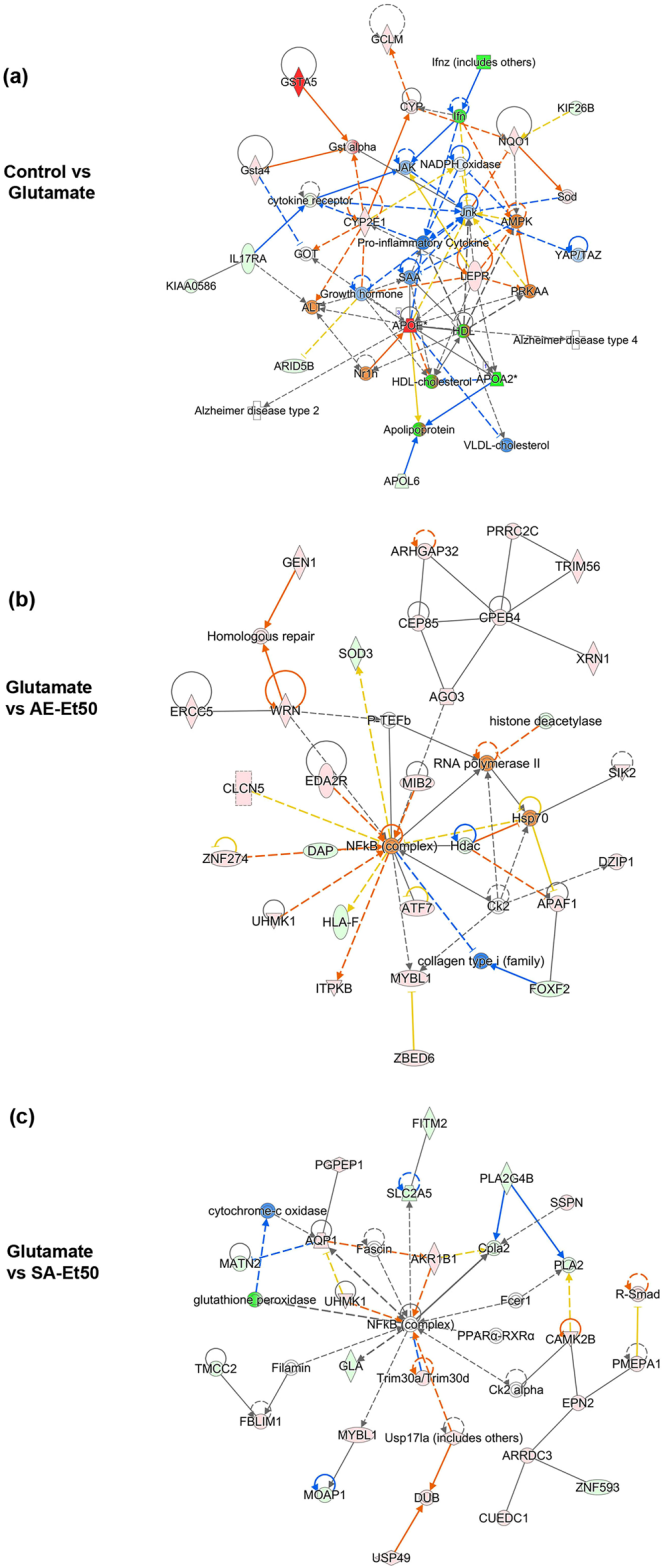
Network analyses of DEGs among treatment conditions in HT22 cells. Graphical representation of the interactions between DEGs identified from cells treated with glutamate (**a**), glutamate with AE extract (**b**), and glutamate with SA extract (**c**). Red, up-regulation; green, down-regulation.

Biological networks of DEGs were also predicted using IPA software. A representative interactome network of DEGs between control and glutamate treatments showed associations with several molecules including “pro-inflammatory cytokine”, “cytokine receptor”, “growth hormone”, and “apolipoprotein” (Fig. 4a). We found that “Alzheimer’s disease” was present among the diseases/disorders significantly associated with DEGs induced by glutamate treatment. In addition, canonical pathways, including “neuroinflammation signaling pathway”, “NF-κB signaling”, “apoptosis signaling”, “autophagy”, and “synaptogenesis signaling pathway” were also significantly associated with DEGs (Fig. 4a).

The interactome network of DEGs from cells co-treated with AE extracts showed associations with several diseases/disorders and canonical pathways, including “central nervous system cancer”, “congenital anomaly of central nervous system”, “apoptosis of neural precursor cells”, “neuroinflammation signaling pathway”, “NF-κB signaling”, and “amyloid processing” (Fig. 4b). The interactome network of DEGs from cells co-treated with SA extracts showed associations with several diseases/disorders and canonical pathways, including “neurovascular coupling signaling pathway”, “neuroinflammation signaling pathway”, “NF-κB signaling”, “amyloid processing”, “synaptic long-term depression”, and “Parkinson’s signaling” (Fig. 4c). Interestingly, the hub gene in the interactome network of DEGs from cells co-treated with AE extracts and cells co-treated with SA extracts was “NF-κB”, which is the key gene responsible for inflammation and several neurodegenerative diseases (Fig. 4b, c).

### RT-qPCR validation of selected DEGs from all treatment conditions

**Figure 5:**
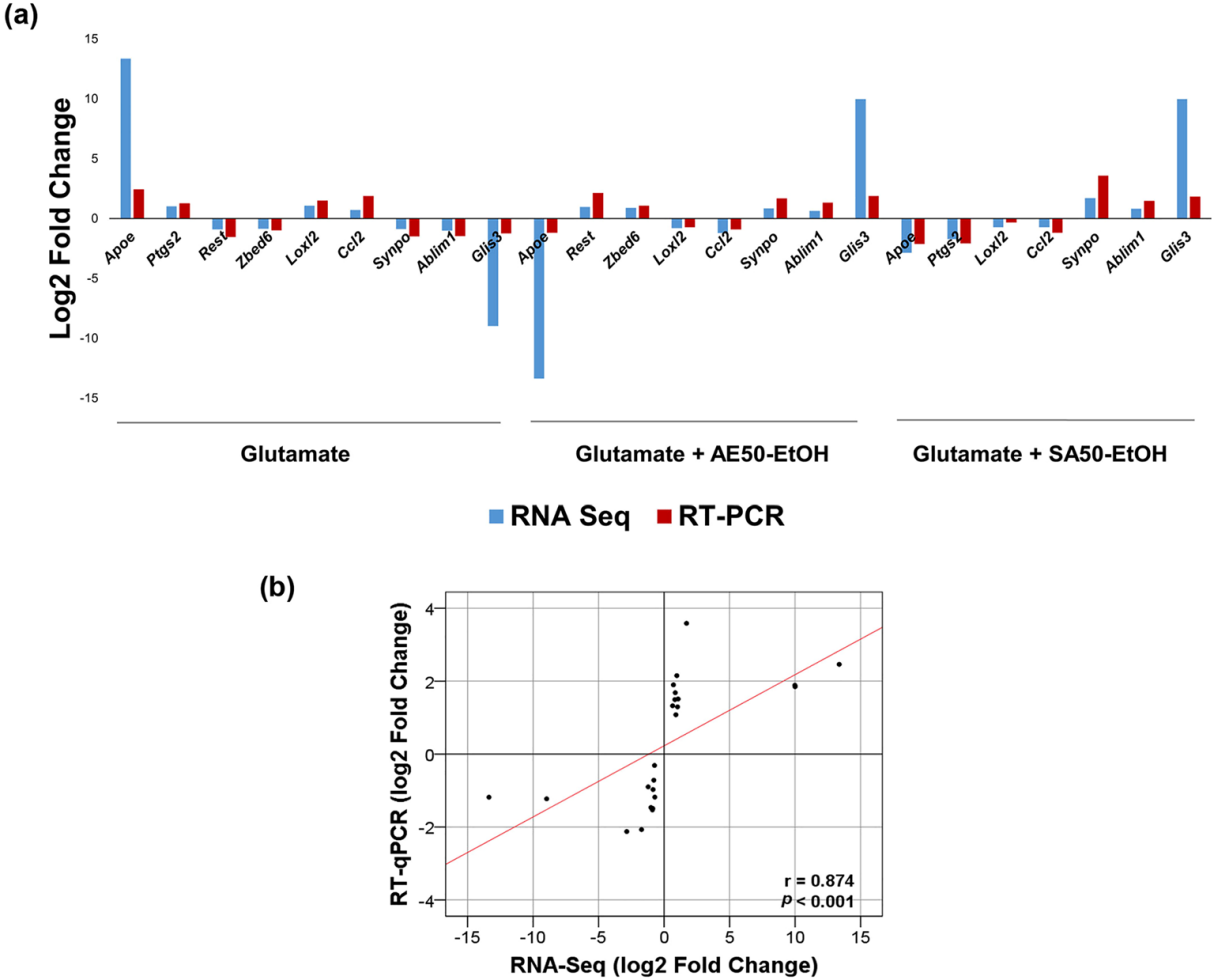
Correlation between gene expression ratios obtained from the transcriptome data and RT-qPCR data. (**a**) Expression levels obtained from transcriptome data (blue) and RT-qPCR data (red). (**b**) Correlation analysis between the transcriptome and RT-qPCR data. Each point represents a value of the log2 of the relative fold change between pairs of treatment-comparison groups (glutamate treated cells vs. vehicle control, glutamate with AE extracts vs. glutamate treated cells, glutamate with SA extracts vs. glutamate treated cells, respectively). Regression line and Spearman’s correlation coefficient are shown.

To validate the reliability of the transcriptome sequencing data, the relative expression levels of 9 DEGs involved in Alzheimer’s disease (*Apoe, Ptgs2, Rest, Zbed6, Loxl2, Ccl2, Synpo, Ablim1,* and *Glis3*; see also Table 2) were analyzed by quantitative RT-qPCR. RNA-seq analysis and RT-qPCR produced similar gene expression profiles (Fig. 5a). A strong correlation between the log2 fold change of the two methods was observed (Spearman’s correlation coefficient = 0.874, *P* < 0.001) (Fig. 5b).

**Table 2:** List of DEGs analyzed in RT-qPCR and their reported changes in association with AD.

Gene		Changes observed in AD patients
*Apoe*	Apolipoprotein E	Upregulation in AD brain^16^
*Ptgs2*	Prostaglandin-endoperoxide synthase 2 (cyclooxygenase 2)	Upregulation in AD brain^17^
*Rest*	Repressor element 1 silencing transcription factor (neuron-restrictive silencer factor)	Downregulation in AD brain^18,19^
*Zbed6*	Zinc finger BED domain-containing protein 6	Upregulation in blood sample^20^
*Loxl2*	Lysyl oxidase-like 2	Upregulation in AD brain^21^
*Ccl2*	Chemokine C–C motif ligand-2 (monocyte chemotactic protein-1)	Upregulation in AD brain and CSF of AD patients^22,23^
*Synpo*	Synaptopodin	Downregulation in AD brain^24^
*Ablim1*	Actin-binding LIM protein 1	AD-risk associated SNPs (rs6646, rs727532) in AD brain^25^
*Glis3*	GLIS Family Zinc Finger 3	AD-risk associated SNPs (rs514716) in CSF of AD patients^26^

**Figure 6:**
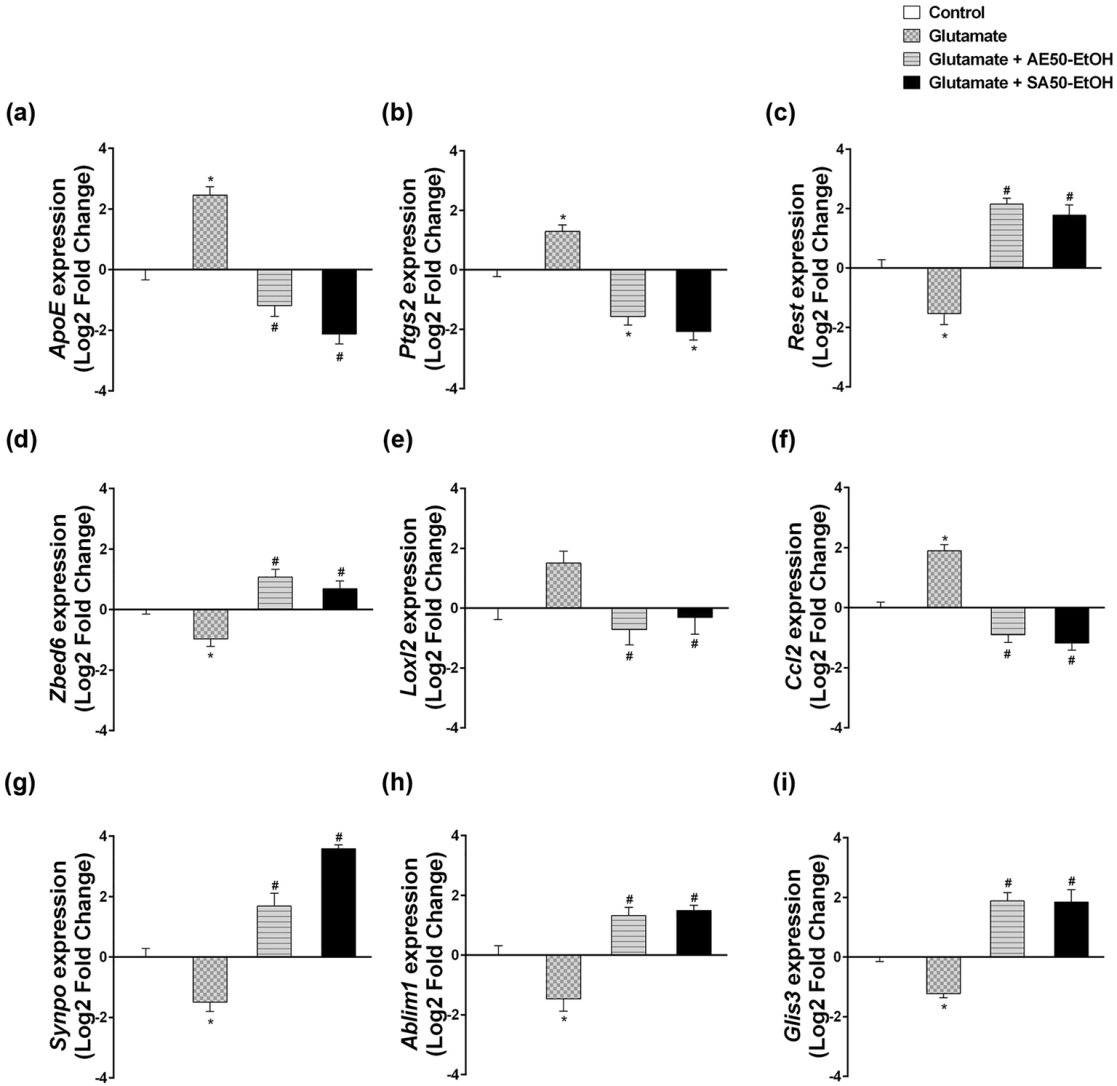
The expression levels of 9 selected DEGs associated with brain function and/or AD pathogenesis determined by RT-PCR for validation of RNA-seq data. Relative expression levels were calculated using the delta-delta threshold cycle (Ct) method and ß-actin as the reference gene. The results are expressed as the mean ± SE, n = 3. *P* < 0.05 is considered significant. *P < 0.05 glutamate treated cells vs. vehicle control, ^#^*P* < 0.05 glutamate with extracts vs. glutamate treated cells.

In RT-qPCR experiment, we found that *Apoe*, *Ptgs2* and *Ccl2* expression was significantly increased while *Loxl2* tended to increase in glutamate treatment compared with controls (*P* < 0.05; Fig. 6a, b, e, f). Co-treatment with AE extract and SA extract significantly attenuated expression of these genes. In addition, *Rest, Zbed6, Synpo, Ablim1,* and *Glis3* expression was significantly decreased in glutamate-treated cells compared with controls (*P* < 0.05; Fig. 6c, d, g, h, i). Moreover, we found that co-treatment with AE extract and SA extract both significantly induced expression of these genes.

## Discussion

In this study we investigated whether glutamate exposure alters the transcriptome profiles in HT22 cells. We conducted an RNA-seq analysis of HT22 cells treated with glutamate, glutamate with AE extract, and glutamate with SA extract. The list of differentially expressed genes (DEGs) from RNA-seq analysis were analyzed using IPA software to predict canonical pathways, diseases/disorders, biological functions, and biological networks significantly associated with DEGs. The results from our study revealed that DEGs induced by glutamate treatment were significantly associated with several neurological diseases and nervous system functions. Moreover, DEGs induced by glutamate treatment were associated with several nervous system canonical pathways including the activation state of Neurovascular Coupling Signaling Pathway and the inhibition state of Neuroinflammation Signaling Pathway and Synaptogenesis Signaling Pathway. We found that the biological network predicted by IPA software of DEGs between control and glutamate treatment showed associations with Alzheimer's disease and several canonical pathways, including “neuroinflammation signaling pathway”, “NF-κB signaling”, “apoptosis signaling”, “autophagy”, and “synaptogenesis signaling pathway”. Interestingly, a hub gene in the interactome network generated using DEGs between control and glutamate treatment was “*Apoe*”, which is the key gene responsible for Alzheimer’s disease.

When HT22 cells were co-treated with glutamate and AE extracts, DEGs were significantly associated with several neurological diseases, including “early-onset Alzheimer disease”. We found that DEGs from cells co-treated with either AE extract or SA extract were significantly associated with nervous system function, including “development of neurons”. Our results revealed that DEGs from cells co-treated with AE extract were associated with the canonical pathways such as the activation state of Natural Killer Cell Signaling Pathway and p53 Signaling Pathway. Interestingly, Co-treatment HT22 cells with AE extract alters the DEGs such as SOD3, which has been found to ameliorate Aβ-induced oxidative damage in neuroblastoma cells^27^, and APAF1, which is associated with apoptotic stimuli and neurodegenerative inducers such as Aβ peptide in Alzheimer's disease^28^. The DEGs from cells co-treated with SA extract were found associated with the canonical pathways such as the activation state of Synaptogenesis Signaling Pathway and Neuroinflammation Signaling Pathway and the inhibition state of Neurovascular Coupling Signaling Pathway. Interestingly, Co-treatment HT22 cells with SA extract alters the DEGs in the Synaptogenesis Signaling Pathway, including APOE.

In addition, the interactome network of DEGs from cells co-treated with AE extract and SA extract showed associations with “neuroinflammation signaling pathway”, “NF-κB signaling”, and “amyloid processing”. Interestingly, we found that a hub gene in the interactome networks generated using DEGs of glutamate-treated HT22 cells co-treated with either AE extract or SA extract was NF-κB, which is a key gene that plays important roles in regulating inflammation, oxidative stress and apoptosis. Neuroinflammatory cytokines are known to reduce the efflux transport of Aβ, thus leading to elevated Aβ concentrations in the brain and increased susceptibility to AD^29^. NF-κB is currently considered an important agent related to the chronic neuroinflammatory state that persists in the AD brain^30,31^. An increased proportion of neurons with nuclear p65, one of the five components that form the NF-κB transcription factor family and a parameter for NF-κB activation, has been observed in the hippocampus and cortex around Aβ plaques in postmortem AD tissues^32^. These findings indicate that AE and SA extracts may exert their effects on neuroinflammation.

The expression levels of DEGs across treatment conditions were determined by RT-PCR for validation of RNA-seq data. Gene expression profiles from RT-PCR results were consistent with the transcriptome data. The expression levels of *Apoe*, *Ptgs2*, and *Ccl2* were significantly increased and *Loxl2* tended to increase in glutamate treatment compared with controls. This is consistent with the changes in these molecules in the AD brain (Table 2). Apolipoprotein E (encoded by *Apoe* gene) is the major genetic risk factor for AD, especially the *Apoe*4 isoform, and it was found that *Apoe* mRNA levels were increased in the brains of patients with AD^16^. *Apoe* expression level is also involved in amyloid accumulation, tau pathology, neurotoxicity, oxidative stress, and neuroinflammation^33,34^. Prostaglandin-endoperoxide synthase 2 (PTGS2), also known as cyclooxygenase 2 (COX2), has been shown to be commonly expressed in the inflammatory cells of the central nervous system. The gene coding PTGS2 is located to 1q31.1 between two regions which are genetic linkage to AD^35^. In addition, elevated PTGS2 expression was found in neuronal cells of the AD brain^17^. Lysyl oxidase-like 2 (LOXL2) plays a role in the pathogenesis of brain malignancy. LOXL2 expression indicated poor overall survival of glioma patients, and LOXL2 may serve as a promising therapeutic target in the treatment of glioma^36,37^. Upregulation of LOX has been associated with Alzheimer’s and non-Alzheimer’s dementia^21^. The chemokine C–C motif ligand-2 (CCL2), also known as monocyte chemotactic protein-1 (MCP-1), is present in the brain and produced by microglia, neurons, activated astrocytes, and mononuclear phagocytes. It was found that CCL2 expression was upregulated in AD brain tissue^22^. Moreover, increased cerebrospinal fluid (CSF) levels of CCL2 have been detected in patients with AD^23^. In this study, we found that HT22 cells co-treated with glutamate in combination with either AE extract or SA extract significantly decreased the expression of these genes.

In addition, *Rest, Zbed6, Synpo, Ablim1,* and *Glis3* expression were significantly decreased in HT22 cells with glutamate treatment compared with vehicle controls. This is relatively consistent with the changes in these molecules in the AD brain except *Zbed6* gene expression (Table 2). Nevertheless, the study led by Naughton et al. 2015 has investigated the expression of *Zbed6* gene in the blood sample of AD patient, whereas in the present study that expression was determined in neuronal cell line. Repressor element 1 silencing transcription factor (REST; also known as neuron-restrictive silencer factor, NRSF) is an essential transcriptional repressor that targets neuron-specific genes^18^. Nuclear translocation of neuronal REST has been shown to be neuroprotective in a healthy aging brain, whereas REST is markedly reduced in AD in vulnerable neuronal populations^18,19^. Zinc finger BED domain-containing protein 6 (ZBED6) is an important transcription factor in placental mammals, affecting development, cell proliferation, and growth^38^. It was found that ZBED6 negatively regulates neuronal differentiation^39^. Synaptopodin (SYNPO) is an actin-associated protein that has been implicated in the structural and functional plasticity of dendritic spines^40^. Interestingly, SYNPO expression in the hippocampus is downregulated in patients with AD^24^. Actin-binding LIM protein 1 (ABLIM1), a member of the LIM-domain protein family, is expressed mainly in the retina, brain, and muscle^41^. ABLIM1 mediates interactions between actin filaments and cytoplasmic targets. It has been shown that ABLIM1 also regulates receptor activator of NF-κB ligand-mediated osteoclast differentiation and motility^42^. SNPs of ABLIM1 have been shown as late-onset Alzheimer disease (LOAD)-susceptibility loci^25^. GLIS Family Zinc Finger 3 (GLIS3) belongs to a family of transcription factors, the Krüppel-like zinc finger proteins^43^. GLIS3 has been associated with an increased risk of diabetes, glaucoma, and neurological disorders including Alzheimer’s disease^44,45^. GLIS3 was found to affect tau levels and Alzheimer’s risk. Genome wide association studies indicated that SNPs (rs514716), located at 9p24.2 in an intron of GLIS3 has been shown significant association with both CSF tau and p-tau levels of AD cases^26^. In this study, we found that co-treatment with glutamate and AE or SA extracts significantly induced the expression of these genes. These findings indicate that glutamate treatment of HT22 cells dysregulated AD-related genes and that both AE and SA extracts reverse some of the glutamate-induced expression changes in these genes. By so doing, these extracts may counter the injurious effect of glutamate on HT22 cells, especially by inducing neuroprotection and inflammation-associated genes. Further investigations on the identified molecular signatures in the canonical pathways (Fig. 3) with their functions associated to AD are recommended. Some potentially interesting targets in response to SA extract include the cAMP Response Element-Binding Protein (CREB) signaling pathway and the neurovascular coupling signaling which were found in activated and inhibited states, respectively. The potential targets in the natural killer cell and p53 signaling pathways were also highlighted for the next study to explore AE extract's action mechanism.

Overall, the findings from our in vitro study in HT22 cells indicate that exposure to high level of glutamate may increase risk of AD by disrupting the expression profiles of AD-related genes, thereby contributing to AD susceptibility. These results raise the relevance and importance of using HT22 cells for AD-related studies, particularly in disease pathogenesis and preclinical development of therapeutics. However, despite the benefits of this cell line, there are also some limitations or disadvantages that should be considered^9^. The HT22 cells are of mouse origin and have no expression of functional *N*-methyl-d-aspartate (NMDA) glutamate receptor, which is known to be expressed in human neurons, thereby the study of glutamate toxicity in this cell line does not cover the receptor-mediated effects as occurred in human cells^9^. Interestingly, this study shows that the role of AE and SA extracts in the inhibition of glutamate-induced HT22 neurotoxicity is, at least in part, mediated by reversing the expression of AD-related genes in the glutamate treatment model. In conclusion, the present study provides newly identified AD-specific molecular signatures in glutamate-injured HT22 cells, suggesting that this model system can be a valuable tool for the screening and evaluation of new anti-AD agents, particularly from natural products.

## Supplementary Information

Supplementary Information.

## Data Availability

The data generated or analyzed during the current study that are relevant to the results presented are included in this article and its Supplementary Information files. Other data that were not relevant to the results presented here are available from the corresponding authors on reasonable request.

## Abbreviations

AChE: Acetylcholinesterase
AD: Alzheimer’s disease
AE: *Acanthus ebracteatus* Vahl
CNS: Central nervous system
DEGs: Differentially expressed genes
DMEM: Dulbecco's modified Eagle medium
FBS: fetal bovine serum
FDR: False discovery rate
IPA: Ingenuity Pathway Analysis
NDDs: Neurodegenerative disorders
NF-κB: Nuclear factor kappa B
RNA-Seq: RNA sequencing
RT-qPCR: real-time quantitative polymerase chain reaction
SA: *Streblus asper* Lour
