# Supplementary material for: Transcriptomic analysis of glutamate-induced HT22 neurotoxicity as a model for screening anti-Alzheimer’s drugs
Source: Sci Rep. 2023 May 4;13:7225. doi: 10.1038/s41598-023-34183-y (PMC10160028; doi:10.1038/s41598-023-34183-y)
Supplement: Supplementary file 1 — Supplementary Information. [file 41598_2023_34183_MOESM1_ESM.docx]

**Supplementary Materials**

**Transcriptomic analysis of glutamate-induced HT22 neurotoxicity as a model for screening anti-Alzheimer’s drugs**


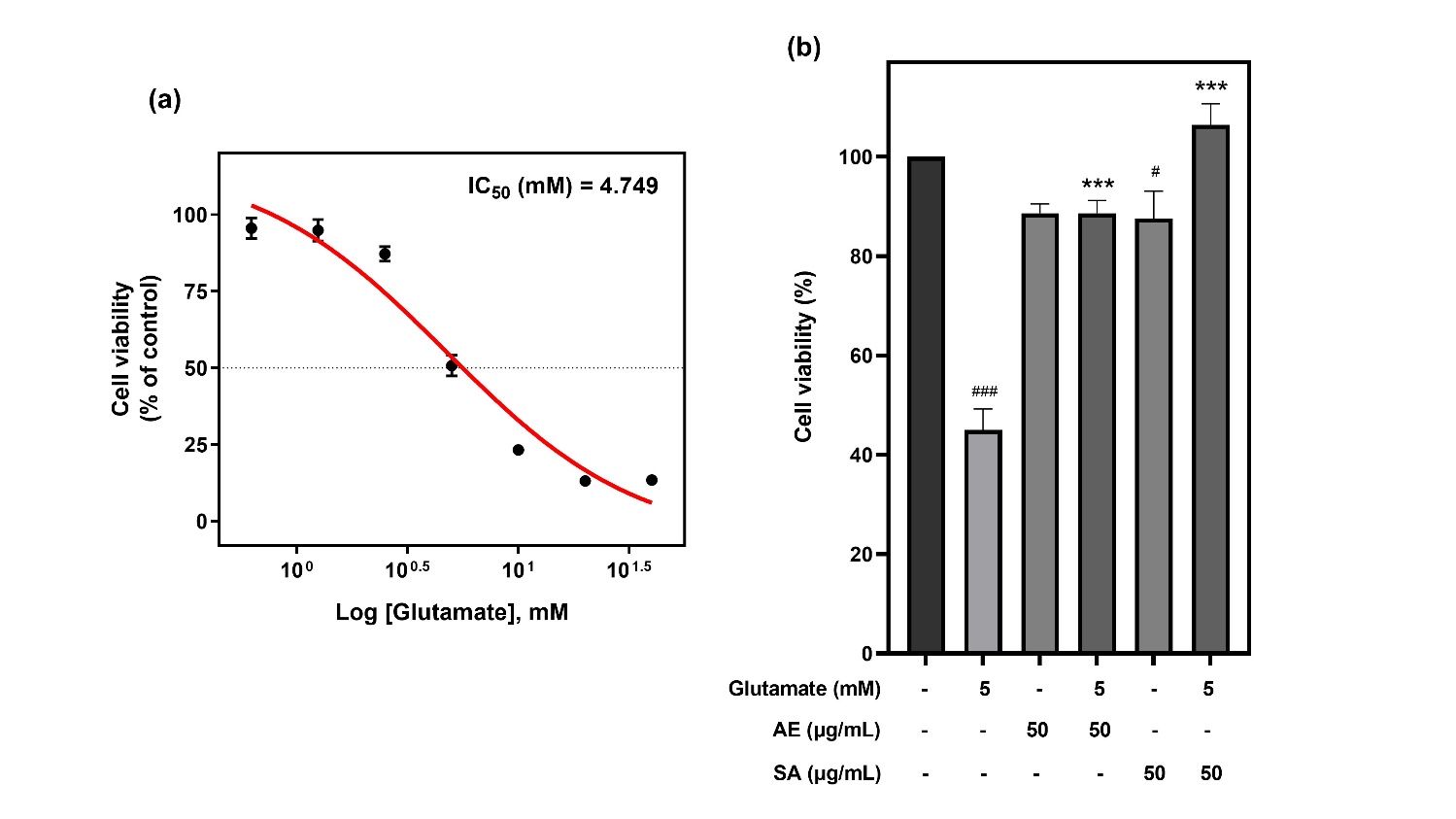


**Supplementary Fig. S1** Effects of glutamate and natural herbal extracts in cultured HT22 cells. Cell viability evaluated by MTT assay after 24 h of treatment confirming (a) the cytotoxic effect of glutamate and (b) the protective effects of co-treatment with AE or SA extract on glutamate-induced cytotoxicity (modified from our previous reports (Prasansuklab et al. 2017; 2018)). Data are expressed as mean ± SD, *^#^P* < 0.05, *^###^P* < 0.001 vs. untreated control; ^🞲🞲🞲^*P* < 0.001 vs. glutamate (5 mM).

**Supplementary Table S1** The list of genes which were differentially expressed in glutamate treatment compared with control cells.

| **geneID** | **Symbol** | **Description** | **Expr Log Ratio** |
| --- | --- | --- | --- |
| NM_001305550 | - | - | -13.9522 |
| 100189605 | Mup19 | major urinary protein 19 | -13.1737 |
| 100039150 | Mup15 | major urinary protein 15 | -13.0885 |
| 21822 | Tgtp1 | T cell specific GTPase 1 | -12.2963 |
| 627914 | Gm14430 | predicted gene 14430 | -12.1892 |
| 546723 | Ppp1r2-ps3 | protein phosphatase 1, regulatory (inhibitor) subunit 2, pseudogene 3 | -12.0634 |
| NR_131218 | - | - | -11.8337 |
| 100039467 | Gm10487 | predicted gene 10487 | -11.5793 |
| NM_001305677 | - | - | -11.2408 |
| 545645 | Gm13283 | predicted gene 13283 | -11.0901 |
| 78655 | Eif3j1 | eukaryotic translation initiation factor 3, subunit J1 | -10.8533 |
| 102238433 | Jmjd7-pla2g4b | predicted gene, 28042 | -10.6618 |
| NM_001305843 | - | - | -10.6618 |
| 100039192 | Tmem254c | transmembrane protein 254c | -10.5603 |
| 100041678 | Gm3500 | predicted gene 3500 | -10.5118 |
| NM_001305632 | - | - | -9.81378 |
| 100504486 | Gdap10 | ganglioside-induced differentiation-associated-protein 10 | -9.29921 |
| 664779 | Zfp91Cntf | Zfp91-Cntf readthrough transcript (NMD candidate) | -8.99435 |
| 226075 | Glis3 | GLIS family zinc finger 3 | -8.96578 |
| 246727 | Oas3 | 2'-5' oligoadenylate synthetase 3 | -2.7866 |
| 11695 | Alx4 | aristaless-like homeobox 4 | -2.71621 |
| 74953 | 4930483K19Rik | RIKEN cDNA 4930483K19 gene | -2.68326 |
| 53374 | Chst3 | carbohydrate (chondroitin 6/keratan) sulfotransferase 3 | -2.40599 |
| 71913 | Tmem79 | transmembrane protein 79 | -2.35364 |
| 71481 | Alpk1 | alpha-kinase 1 | -2.0163 |
| 100134861 | ChkbCpt1b | Chkb-Cpt1b readthrough transcript (NMD candidate) | -1.85244 |
| 69772 | Bdh2 | 3-hydroxybutyrate dehydrogenase, type 2 | -1.83503 |
| 75029 | Purg | purine-rich element binding protein G | -1.78701 |
| 21897 | Tlr1 | toll-like receptor 1 | -1.73434 |
| 16876 | Lhx9 | LIM homeobox protein 9 | -1.62149 |
| 319586 | Celf5 | CUGBP, Elav-like family member 5 | -1.59296 |
| 14536 | Nr6a1 | nuclear receptor subfamily 6, group A, member 1 | -1.5755 |
| 677884 | Palm2Akap2 | paralemmin A kinase anchor protein | -1.53903 |
| 11287 | Pzp | pregnancy zone protein | -1.5119 |
| 11816 | Apoe | apolipoprotein E | -1.48817 |
| 69629 | 2310039L15Rik | RIKEN cDNA 2310039L15 gene | -1.38422 |
| 71939 | Apol6 | apolipoprotein L 6 | -1.30058 |
| 11754 | Aoc3 | amine oxidase, copper containing 3 | -1.28591 |
| 216439 | Agap2 | ArfGAP with GTPase domain, ankyrin repeat and PH domain 2 | -1.27785 |
| 100861615 | LOC100861615 | alpha takusan-like | -1.22239 |
| 16558 | Kif16b | kinesin family member 16B | -1.21775 |
| 79221 | Hdac9 | histone deacetylase 9 | -1.20645 |
| 208431 | Shroom4 | shroom family member 4 | -1.19265 |
| 213081 | Wdr19 | WD repeat domain 19 | -1.17526 |
| 100101807 | 1700047I17Rik2 | RIKEN cDNA 1700047I17 gene 2 | -1.14564 |
| 330267 | Thsd7a | thrombospondin, type I, domain containing 7A | -1.11905 |
| 16589 | Uhmk1 | U2AF homology motif (UHM) kinase 1 | -1.11032 |
| 14367 | Fzd5 | frizzled homolog 5 (Drosophila) | -1.0679 |
| 100039934 | Gm15085 | predicted gene 15085 | -1.06031 |
| 16656 | Hivep3 | human immunodeficiency virus type I enhancer binding protein 3 | -1.02885 |
| 170829 | Tram2 | translocating chain-associating membrane protein 2 | -1.01162 |
| 226251 | Ablim1 | actin-binding LIM protein 1 | -1.00277 |
| 17101 | Lyst | lysosomal trafficking regulator | -0.98844 |
| 329828 | AI464131 | expressed sequence AI464131 | -0.97167 |
| 269152 | Kif26b | kinesin family member 26B | -0.96321 |
| 23805 | Apc2 | adenomatosis polyposis coli 2 | -0.93198 |
| 333789 | N4bp2 | NEDD4 binding protein 2 | -0.93074 |
| 19712 | Rest | RE1-silencing transcription factor | -0.88967 |
| 12728 | Clcn5 | chloride channel 5 | -0.88786 |
| 24127 | Xrn1 | 5'-3' exoribonuclease 1 | -0.88104 |
| 170753 | Zfp704 | zinc finger protein 704 | -0.8807 |
| 20363 | Sepp1 | selenoprotein P, plasma, 1 | -0.88042 |
| 104027 | Synpo | synaptopodin | -0.87162 |
| 59038 | Pxmp4 | peroxisomal membrane protein 4 | -0.87054 |
| 19714 | Rev3l | REV3-like, catalytic subunit of DNA polymerase zeta RAD54 like (S. cerevisiae) | -0.86802 |
| 224093 | Fam43a | family with sequence similarity 43, member A | -0.8489 |
| 17920 | Myo6 | myosin VI | -0.84876 |
| 11504 | Adamts1 | a disintegrin-like and metallopeptidase (reprolysin type) with thrombospondin type 1 motif, 1 | -0.84347 |
| 667118 | Zbed6 | zinc finger, BED domain containing 6 | -0.84194 |
| 80285 | Parp9 | poly (ADP-ribose) polymerase family, member 9 | -0.83894 |
| 52850 | Sgsm1 | small G protein signaling modulator 1 | -0.83463 |
| 57435 | Plin4 | perilipin 4 | -0.82704 |
| 17268 | Meis1 | Meis homeobox 1 | -0.82312 |
| 665211 | Gm14326 | predicted gene 14326 | -0.82038 |
| 72723 | Zfp74 | zinc finger protein 74 | -0.81614 |
| 224836 | Usp49 | ubiquitin specific peptidase 49 | -0.81302 |
| 442834 | D830031N03Rik | RIKEN cDNA D830031N03 gene | -0.80539 |
| 240476 | Zfp407 | zinc finger protein 407 | -0.79564 |
| 241062 | Pgap1 | post-GPI attachment to proteins 1 | -0.79355 |
| 18596 | Pdgfrb | platelet derived growth factor receptor, beta polypeptide | -0.78716 |
| 474332 | Dnm3os | dynamin 3, opposite strand | -0.78003 |
| 63953 | Dusp10 | dual specificity phosphatase 10 | -0.77239 |
| 71371 | Arid5b | AT rich interactive domain 5B (MRF1-like) | -0.76876 |
| 75815 | 4930470H14Rik | RIKEN cDNA 4930470H14 gene | -0.7679 |
| 320871 | B230206H07Rik | RIKEN cDNA B230206H07 gene | -0.76338 |
| 268980 | Strn | striatin, calmodulin binding protein | -0.76234 |
| 100416706 | AA987161 | expressed sequence AA987161 | -0.75052 |
| 50706 | Postn | periostin, osteoblast specific factor | -0.74983 |
| 109181 | Trip11 | thyroid hormone receptor interactor 11 | -0.74688 |
| 71710 | Lrrcc1 | leucine rich repeat and coiled-coil domain containing 1 | -0.74472 |
| 77976 | Nuak1 | NUAK family, SNF1-like kinase, 1 | -0.73517 |
| 230895 | Vps13d | vacuolar protein sorting 13 D (yeast) | -0.73073 |
| 268396 | Sh3pxd2b | SH3 and PX domains 2B | -0.73027 |
| 12704 | Cit | citron | -0.73008 |
| 227059 | Slc39a10 | solute carrier family 39 (zinc transporter), member 10 | -0.72676 |
| 20350 | Sema3f | sema domain, immunoglobulin domain (Ig), short basic domain, secreted, (semaphorin) 3F | -0.72238 |
| 12288 | Cacna1c | calcium channel, voltage-dependent, L type, alpha 1C subunit | -0.71961 |
| 433938 | Mn1 | meningioma 1 | -0.71864 |
| 238123 | Cog5 | component of oligomeric golgi complex 5 | -0.71414 |
| 18986 | Pou2f1 | POU domain, class 2, transcription factor 1 | -0.70834 |
| 104799 | Vipas39 | VPS33B interacting protein, apical-basolateral polarity regulator, spe-39 homolog | -0.70066 |
| 21961 | Tns1 | tensin 1 | -0.70032 |
| 223922 | Atf7 | activating transcription factor 7 | -0.69927 |
| 99382 | Abtb2 | ankyrin repeat and BTB (POZ) domain containing 2 | -0.69648 |
| 12825 | Col3a1 | collagen, type III, alpha 1 | -0.69565 |
| 74202 | Fblim1 | filamin binding LIM protein 1 | -0.69403 |
| 76954 | St5 | suppression of tumorigenicity 5 | -0.68934 |
| 16172 | Il17ra | interleukin 17 receptor A | -0.68749 |
| 13822 | Epb4.1l2 | erythrocyte protein band 4.1-like 2 | -0.68343 |
| 16177 | Il1r1 | interleukin 1 receptor, type I | -0.68335 |
| 68732 | Lrrc16a | leucine rich repeat containing 16A | -0.6811 |
| 667410 | Gm8615 | glucosamine-6-phosphate deaminase 1 pseudogene | -0.67972 |
| 19300 | Abcd4 | ATP-binding cassette, sub-family D (ALD), member 4 | -0.67919 |
| 15273 | Hivep2 | human immunodeficiency virus type I enhancer binding protein 2 | -0.67807 |
| 23794 | Adamts5 | a disintegrin-like and metallopeptidase (reprolysin type) with thrombospondin type 1 motif, 5 (aggrecanase-2) | -0.67794 |
| 22288 | Utrn | utrophin | -0.67774 |
| 77057 | Ston1 | stonin 1 | -0.67619 |
| 20847 | Stat2 | signal transducer and activator of transcription 2 | -0.67357 |
| 210094 | Iglon5 | IgLON family member 5 | -0.66941 |
| 76967 | 2700049A03Rik | RIKEN cDNA 2700049A03 gene | -0.66879 |
| 77578 | Bcl9 | B cell CLL/lymphoma 9 | -0.66819 |
| 17175 | Masp2 | mannan-binding lectin serine peptidase 2 | -0.66476 |
| 15530 | Hspg2 | perlecan (heparan sulfate proteoglycan 2) | -0.66119 |
| 71085 | Arhgap19 | Rho GTPase activating protein 19 | -0.65852 |
| 213556 | Plekhh2 | pleckstrin homology domain containing, family H (with MyTH4 domain) member 2 | -0.65754 |
| 11603 | Agrn | agrin | -0.65541 |
| 319880 | Tmcc3 | transmembrane and coiled coil domains 3 | -0.6531 |
| 12842 | Col1a1 | collagen, type I, alpha 1 | -0.65277 |
| 11735 | Ank3 | ankyrin 3, epithelial | -0.65016 |
| 320634 | Ocrl | oculocerebrorenal syndrome of Lowe | -0.64642 |
| 12442 | Ccnb2 | cyclin B2 | -0.64557 |
| 16971 | Lrp1 | low density lipoprotein receptor-related protein 1 | -0.64505 |
| 16599 | Klf3 | Kruppel-like factor 3 (basic) | -0.64449 |
| 16859 | Lgals9 | lectin, galactose binding, soluble 9 | -0.6439 |
| 277973 | Slc9a5 | solute carrier family 9 (sodium/hydrogen exchanger), member 5 | -0.64274 |
| 24063 | Spry1 | sprouty homolog 1 (Drosophila) | -0.64088 |
| 12837 | Col8a1 | collagen, type VIII, alpha 1 | -0.63966 |
| 66573 | Dzip1 | DAZ interacting protein 1 | -0.63714 |
| 70573 | Tbccd1 | TBCC domain containing 1 | -0.63702 |
| 21826 | Thbs2 | thrombospondin 2 | -0.63287 |
| 70012 | Cep85 | centrosomal protein 85 | -0.62904 |
| 231861 | Tnrc18 | trinucleotide repeat containing 18 | -0.62722 |
| 73173 | Pcdh18 | protocadherin 18 | -0.62085 |
| 70717 | Medag | mesenteric estrogen dependent adipogenesis | -0.62057 |
| 320528 | Vps13c | vacuolar protein sorting 13C (yeast) | -0.61949 |
| 235461 | Fam63b | family with sequence similarity 63, member B | -0.61901 |
| 77097 | Tanc2 | tetratricopeptide repeat, ankyrin repeat and coiled-coil containing 2 | -0.61829 |
| 21951 | Tnks | tankyrase, TRF1-interacting ankyrin-related ADP-ribose polymerase | -0.61449 |
| 80288 | Bcl9l | B cell CLL/lymphoma 9-like | -0.61417 |
| 59090 | Midn | midnolin | -0.61366 |
| 381022 | Kmt2d | lysine (K)-specific methyltransferase 2D | -0.61353 |
| 103466 | Nt5dc3 | 5'-nucleotidase domain containing 3 | -0.61022 |
| 235587 | Parp3 | poly (ADP-ribose) polymerase family, member 3 | -0.60994 |
| 72238 | Tbc1d5 | TBC1 domain family, member 5 | -0.60954 |
| 269878 | Megf8 | multiple EGF-like-domains 8 | -0.6084 |
| 22720 | Zfp62 | zinc finger protein 62 | -0.604 |
| 21825 | Thbs1 | thrombospondin 1 | -0.60234 |
| 268970 | Arhgap28 | Rho GTPase activating protein 28 | -0.6022 |
| 214791 | Sertad4 | SERTA domain containing 4 | -0.60095 |
| 14268 | Fn1 | fibronectin 1 | -0.6008 |
| 212427 | A730008H23Rik | RIKEN cDNA A730008H23 gene | -0.59698 |
| 70466 | Ckap2l | cytoskeleton associated protein 2-like | -0.5894 |
| 12834 | Col6a2 | collagen, type VI, alpha 2 | -0.58919 |
| 216856 | Nlgn2 | neuroligin 2 | -0.58911 |
| 14924 | Magi1 | membrane associated guanylate kinase, WW and PDZ domain containing 1 | -0.58893 |
| 78455 | Helz | helicase with zinc finger domain | -0.58669 |
| 382090 | Cep162 | centrosomal protein 162 | -0.58614 |
| 209683 | Ttc28 | tetratricopeptide repeat domain 28 | -0.58496 |
| 15042 | H2-T24 | histocompatibility 2, T region locus 24 | 0.589888 |
| 50877 | Neu3 | neuraminidase 3 | 0.591007 |
| 18104 | Nqo1 | NAD(P)H dehydrogenase, quinone 1 | 0.594075 |
| 16151 | Ikbkg | inhibitor of kappaB kinase gamma | 0.596413 |
| 72612 | 2700029M09Rik | RIKEN cDNA 2700029M09 gene | 0.597121 |
| 30057 | Timm8b | translocase of inner mitochondrial membrane 8B | 0.60141 |
| 72084 | Pigx | phosphatidylinositol glycan anchor biosynthesis, class X | 0.603011 |
| 93734 | Mpv17l | Mpv17 transgene, kidney disease mutant-like | 0.603666 |
| 195040 | Tmem199 | transmembrane protein 199 | 0.607133 |
| 11688 | Alox8 | arachidonate 8-lipoxygenase | 0.615672 |
| 68209 | Rnaseh2c | ribonuclease H2, subunit C | 0.615833 |
| 22173 | Tyr | tyrosinase | 0.616531 |
| 11958 | Atp5k | ATP synthase, H+ transporting, mitochondrial F1F0 complex, subunit E | 0.617143 |
| 241919 | Slc7a14 | solute carrier family 7 (cationic amino acid transporter, y+ system), member 14 | 0.617299 |
| 13106 | Cyp2e1 | cytochrome P450, family 2, subfamily e, polypeptide 1 | 0.620333 |
| 27047 | Omd | osteomodulin | 0.624506 |
| 70036 | Dancr | differentiation antagonizing non-protein coding RNA | 0.625835 |
| 66950 | Tmem206 | transmembrane protein 206 | 0.628351 |
| 319931 | A330032B11Rik | RIKEN cDNA A330032B11 gene | 0.63427 |
| 56809 | Gmeb1 | glucocorticoid modulatory element binding protein 1 | 0.639753 |
| 211151 | Churc1 | churchill domain containing 1 | 0.665664 |
| 67836 | Wdr83 | WD repeat domain containing 83 | 0.677044 |
| 12765 | Cxcr2 | chemokine (C-X-C motif) receptor 2 | 0.681624 |
| 73102 | Slc22a23 | solute carrier family 22, member 23 | 0.68245 |
| 100040531 | Dynlt1f | dynein light chain Tctex-type 1F | 0.690491 |
| 12447 | Ccne1 | cyclin E1 | 0.690845 |
| 68559 | Pdrg1 | p53 and DNA damage regulated 1 | 0.691558 |
| 68332 | Sdhaf1 | succinate dehydrogenase complex assembly factor 1 | 0.694245 |
| 225994 | Nmrk1 | nicotinamide riboside kinase 1 | 0.696273 |
| 69920 | Polr2i | polymerase (RNA) II (DNA directed) polypeptide I | 0.699283 |
| 320587 | Tmem88b | transmembrane protein 88B | 0.701985 |
| 14630 | Gclm | glutamate-cysteine ligase, modifier subunit | 0.70878 |
| 14319 | Fth1 | ferritin heavy chain 1 | 0.709713 |
| 319996 | Casc4 | cancer susceptibility candidate 4 | 0.714778 |
| 20296 | Ccl2 | chemokine (C-C motif) ligand 2 | 0.717525 |
| 69723 | Rpain | RPA interacting protein | 0.729127 |
| 13885 | Esd | esterase D/formylglutathione hydrolase | 0.734007 |
| 83673 | Snhg1 | small nucleolar RNA host gene 1 | 0.738904 |
| 434768 | Rhox8 | reproductive homeobox 8 | 0.740123 |
| 474160 | BC033916 | cDNA sequence BC033916 | 0.740772 |
| 67739 | Slc48a1 | solute carrier family 48 (heme transporter), member 1 | 0.742411 |
| 71670 | Acy3 | aspartoacylase (aminoacylase) 3 | 0.749137 |
| 195046 | Nlrp1a | NLR family, pyrin domain containing 1A | 0.751087 |
| 433406 | Gm13363 | predicted gene 13363 | 0.753673 |
| 227715 | Exosc2 | exosome component 2 | 0.775913 |
| 382074 | Foxr1 | forkhead box R1 | 0.77826 |
| 209003 | Rbmx2 | RNA binding motif protein, X-linked 2 | 0.7868 |
| 14283 | Fosl1 | fos-like antigen 1 | 0.794227 |
| 68371 | Pbld1 | phenazine biosynthesis-like protein domain containing 1 | 0.798447 |
| 100038608 | Gm10389 | predicted gene 10389 | 0.80101 |
| 11677 | Akr1b3 | aldo-keto reductase family 1, member B3 (aldose reductase) | 0.815356 |
| 109857 | Cbr3 | carbonyl reductase 3 | 0.82216 |
| 108841 | Rdh13 | retinol dehydrogenase 13 (all-trans and 9-cis) | 0.833149 |
| 67731 | Fbxo32 | F-box protein 32 | 0.840801 |
| 17141 | Magea5 | melanoma antigen, family A, 5 | 0.842459 |
| 103784 | Wdr92 | WD repeat domain 92 | 0.858563 |
| 73162 | Otud3 | OTU domain containing 3 | 0.876559 |
| 595136 | Ndufs5 | NADH dehydrogenase (ubiquinone) Fe-S protein 5 | 0.896513 |
| 208501 | 1810043H04Rik | RIKEN cDNA 1810043H04 gene | 0.90209 |
| 100039060 | 0610010B08Rik | RIKEN cDNA 0610010B08 gene | 0.923716 |
| 606735 | A330069E16Rik | RIKEN cDNA A330069E16 gene | 0.930234 |
| 75545 | 1700019B21Rik | RIKEN cDNA 1700019B21 gene | 0.934486 |
| 11670 | Aldh3a1 | aldehyde dehydrogenase family 3, subfamily A1 | 0.954196 |
| 319166 | Hist1h2ae | histone cluster 1, H2ae | 0.961806 |
| 66664 | Tmem41a | transmembrane protein 41a | 0.964317 |
| 22152 | Tubb3 | tubulin, beta 3 class III | 0.967617 |
| 665155 | Srp54b | signal recognition particle 54B | 0.974555 |
| 16847 | Lepr | leptin receptor | 0.97949 |
| 17841 | Mup2 | major urinary protein 2 | 1.002004 |
| 66169 | Tomm7 | translocase of outer mitochondrial membrane 7 homolog (yeast) | 1.002482 |
| 104943 | Fam110c | family with sequence similarity 110, member C | 1.018011 |
| 19225 | Ptgs2 | prostaglandin-endoperoxide synthase 2 | 1.02197 |
| 103711 | Pnpo | pyridoxine 5'-phosphate oxidase | 1.045088 |
| 12068 | Bet1 | blocked early in transport 1 homolog (S. cerevisiae) | 1.065211 |
| 94352 | Loxl2 | lysyl oxidase-like 2 | 1.068809 |
| 664849 | Gm7367 | 1110014K08Rik pseudogene | 1.073415 |
| 14860 | Gsta4 | glutathione S-transferase, alpha 4 | 1.095128 |
| 102465886 | Mir8094 | microRNA mir-8094 | 1.102747 |
| 66300 | Prr24 | InaF motif containing 1 | 1.107092 |
| 73106 | Prss57 | protease, serine 57 | 1.111235 |
| 69576 | Smco1 | single-pass membrane protein with coiled-coil domains 1 | 1.167868 |
| 382245 | Tmem29 | transmembrane protein 29 | 1.21201 |
| 71306 | Mfap3l | microfibrillar-associated protein 3-like | 1.214125 |
| 18682 | Phkg1 | phosphorylase kinase gamma 1 | 1.215243 |
| 67531 | 5730408K05Rik | RIKEN cDNA 5730408K05 gene | 1.218579 |
| 18812 | Prl2c3 | prolactin family 2, subfamily c, member 3 | 1.249879 |
| 70005 | Znf41-ps | ZNF41, pseudogene | 1.424498 |
| 18811 | Prl2c2 | prolactin family 2, subfamily c, member 2 | 1.545295 |
| 68857 | Dtwd2 | DTW domain containing 2 | 1.576575 |
| 24013 | Grk1 | G protein-coupled receptor kinase 1 | 1.954196 |
| 12499 | Entpd5 | ectonucleoside triphosphate diphosphohydrolase 5 | 2.338802 |
| 19018 | Scand1 | SCAN domain-containing 1 | 2.384392 |
| 100306944 | Snora73a | small nucleolar RNA, H/ACA box 73a | 2.423162 |
| 15368 | Hmox1 | heme oxygenase (decycling) 1 | 2.51295 |
| 93712 | Pcdhga4 | protocadherin gamma subfamily A, 4 | 2.608809 |
| 19817 | Rn7sk | RNA, 7SK, nuclear | 2.722057 |
| 14816 | Grm1 | glutamate receptor, metabotropic 1 | 2.722466 |
| 100039054 | Mup12 | major urinary protein 12 | 4.375039 |
| NM_001305675 | - | - | 4.584963 |
| 100039890 | Gm15093 | predicted gene 15093 | 5.906891 |
| 13056 | Cyb561 | cytochrome b-561 | 9.430453 |
| 100859931 | Gm20604 | predicted gene 20604 | 9.994353 |
| 100041515 | Gm3383 | predicted gene 3383 | 10.11634 |
| 100041874 | Gm3558 | predicted gene 3558 | 10.11634 |
| 320333 | D830030K20Rik | RIKEN cDNA D830030K20 gene | 10.24079 |
| NR_131225 | - | - | 10.27612 |
| 211429 | Pla2g4b | phospholipase A2, group IVB (cytosolic) | 10.64386 |
| 100042149 | Gm3696 | predicted gene 3696 | 10.85331 |
| 100039240 | Gm10058 | predicted gene 10058 | 11.18611 |
| 17840 | Mup1 | major urinary protein 1 | 11.90689 |
| NM_001305819 | - | - | 11.94031 |
| NR_131230 | - | - | 12.23182 |
| 14857 | Gsta1 | glutathione S-transferase, alpha 1 (Ya) | 12.34983 |
| 13999 | Gm14288 | predicted gene 14288 | 12.38532 |
| 11807 | Apoa2 | apolipoprotein A-II | 12.40674 |
| 73385 | Fam177a | family with sequence similarity 177, member A | 12.49185 |
| NM_001305844 | - | - | 13.36632 |

**Supplementary Table S2** The list of genes which were differentially expressed in glutamate plus AE extract treatment compared with glutamate treatment HT22 cells.

| **geneID** | **Symbol** | **Description** | **Expr Log Ratio** |
| --- | --- | --- | --- |
| 102465900 | Mir8112 | microRNA 8112 | -14.1033 |
| NM_001305844 | - | - | -13.3663 |
| 664849 | Gm7367 | 1110014K08Rik pseudogene | -13.1469 |
| 100039060 | 0610010B08Rik | RIKEN cDNA 0610010B08 gene | -13.1098 |
| 100039890 | Gm15093 | predicted gene 15093 | -12.9293 |
| 100041230 | Hist1h4m | histone cluster 1, H4m | -12.7078 |
| NR_131230 | - | - | -12.2318 |
| 107885 | Mthfs | 5, 10-methenyltetrahydrofolate synthetase | -12.0835 |
| 100042149 | Gm3696 | predicted gene 3696 | -10.8533 |
| 545007 | Gm5796 | predicted gene 5796 | -10.5118 |
| NR_131225 | - | - | -10.2761 |
| 100041874 | Gm3558 | predicted gene 3558 | -10.1163 |
| 100859931 | Gm20604 | predicted gene 20604 | -9.99435 |
| NM_001305675 | - | - | -9.90689 |
| 74735 | Trim14 | tripartite motif-containing 14 | -9.67948 |
| 19259 | Ptpn5 | protein tyrosine phosphatase, non-receptor type 5 | -9.27612 |
| 13999 | Gm14288 | predicted gene 14288 | -7.0634 |
| 100039054 | Mup12 | major urinary protein 12 | -4.50057 |
| 66330 | 1700020L24Rik | RIKEN cDNA 1700020L24 gene | -3.72437 |
| 12944 | Crp | C-reactive protein, pentraxin-related | -3.27302 |
| 93712 | Pcdhga4 | protocadherin gamma subfamily A, 4 | -2.60881 |
| 12836 | Col7a1 | collagen, type VII, alpha 1 | -2.11548 |
| 213236 | Dnd1 | dead end homolog 1 (zebrafish) | -2.07276 |
| 319191 | Hist1h2ai | histone cluster 1, H2ai | -2.0358 |
| 21646 | Tcte2 | t-complex-associated testis expressed 2 | -1.92093 |
| 69994 | Rsc1a1 | regulatory solute carrier protein, family 1, member 1 | -1.89077 |
| 319152 | Hist1h3h | histone cluster 1, H3h | -1.73631 |
| 15957 | Ifit1 | interferon-induced protein with tetratricopeptide repeats 1 | -1.73008 |
| 211429 | Pla2g4b | phospholipase A2, group IVB (cytosolic) | -1.6495 |
| 238871 | Pde4d | phosphodiesterase 4D, cAMP specific | -1.58496 |
| 654432 | Gm7334 | B-cell translocation gene 3 pseudogene | -1.57808 |
| 320273 | B230208H11Rik | RIKEN cDNA B230208H11 gene | -1.50649 |
| 11806 | Apoa1 | apolipoprotein A-I | -1.44309 |
| 58229 | Efcc1 | EF hand and coiled-coil domain containing 1 | -1.41954 |
| 223332 | Ranbp3l | RAN binding protein 3-like | -1.41773 |
| 171286 | Slc12a8 | solute carrier family 12 (potassium/chloride transporters), member 8 | -1.41504 |
| 69770 | 1600002K03Rik | RIKEN cDNA 1600002K03 gene | -1.38051 |
| 17116 | Mab21l1 | mab-21-like 1 (C. elegans) | -1.29546 |
| 545652 | Gm13275 | predicted gene 13275 | -1.28011 |
| 71583 | 9130008F23Rik | RIKEN cDNA 9130008F23 gene | -1.27949 |
| 70005 | Znf41-ps | ZNF41, pseudogene | -1.26303 |
| 18811 | Prl2c2 | prolactin family 2, subfamily c, member 2 | -1.26201 |
| 69576 | Smco1 | single-pass membrane protein with coiled-coil domains 1 | -1.21487 |
| 20296 | Ccl2 | chemokine (C-C motif) ligand 2 | -1.21465 |
| 78512 | 3300005D01Rik | RIKEN cDNA 3300005D01 gene | -1.20163 |
| 66300 | Prr24 | InaF motif containing 1 | -1.17798 |
| 621080 | AI429214 | expressed sequence AI429214 | -1.16416 |
| 100504661 | Gm20337 | predicted gene, 20337 | -1.16314 |
| 70348 | Ube2cbp | ubiquitin-conjugating enzyme E2C binding protein | -1.14618 |
| 68857 | Dtwd2 | DTW domain containing 2 | -1.12744 |
| 208501 | 1810043H04Rik | RIKEN cDNA 1810043H04 gene | -1.12152 |
| 99543 | Olfml3 | olfactomedin-like 3 | -1.10193 |
| 72265 | Tram1 | translocating chain-associating membrane protein 1 | -1.05938 |
| 20522 | Slc23a1 | solute carrier family 23 (nucleobase transporters), member 1 | -1.05853 |
| 319166 | Hist1h2ae | histone cluster 1, H2ae | -1.02834 |
| 18612 | Etv4 | ets variant 4 | -1.02415 |
| 14165 | Fgf10 | fibroblast growth factor 10 | -1.00813 |
| 192187 | Stab1 | stabilin 1 | -0.98951 |
| 17112 | Tm4sf1 | transmembrane 4 superfamily member 1 | -0.96528 |
| 69094 | Tmem160 | transmembrane protein 160 | -0.95057 |
| 626832 | Gm6710 | predicted gene 6710 | -0.94571 |
| 108153 | Adamts7 | a disintegrin-like and metallopeptidase (reprolysin type) with thrombospondin type 1 motif, 7 | -0.94397 |
| 77035 | Kdm8 | lysine (K)-specific demethylase 8 | -0.94271 |
| 16323 | Inhba | inhibin beta-A | -0.93217 |
| 382245 | Tmem29 | transmembrane protein 29 | -0.91594 |
| 101544 | Zfp575 | zinc finger protein 575 | -0.9085 |
| 20613 | Snai1 | snail family zinc finger 1 | -0.89648 |
| 327978 | Slfn5 | schlafen 5 | -0.88881 |
| 319171 | Hist1h2ap | histone cluster 1, H2ap | -0.888 |
| 17082 | Il1rl1 | interleukin 1 receptor-like 1 | -0.88492 |
| 381409 | Cdh26 | cadherin-like 26 | -0.87832 |
| 20657 | Sod3 | superoxide dismutase 3, extracellular | -0.87744 |
| 66362 | Exosc3 | exosome component 3 | -0.85824 |
| 56149 | Grasp | GRP1 (general receptor for phosphoinositides 1)-associated scaffold protein | -0.85407 |
| NR_131219 | - | - | -0.82612 |
| 18682 | Phkg1 | phosphorylase kinase gamma 1 | -0.80897 |
| 21923 | Tnc | tenascin C | -0.80869 |
| 192970 | Dhrs11 | dehydrogenase/reductase (SDR family) member 11 | -0.80085 |
| 100040531 | Dynlt1f | dynein light chain Tctex-type 1F | -0.79687 |
| 72269 | Cda | cytidine deaminase | -0.79425 |
| 94352 | Loxl2 | lysyl oxidase-like 2 | -0.78383 |
| 56488 | Nxt1 | NTF2-related export protein 1 | -0.78015 |
| 230752 | Eva1b | eva-1 homolog B (C. elegans) | -0.77859 |
| 64292 | Ptges | prostaglandin E synthase | -0.77487 |
| 11677 | Akr1b3 | aldo-keto reductase family 1, member B3 (aldose reductase) | -0.77183 |
| 230696 | AU022252 | expressed sequence AU022252 | -0.76419 |
| 69694 | Tatdn1 | TatD DNase domain containing 1 | -0.76402 |
| 29817 | Igfbp7 | insulin-like growth factor binding protein 7 | -0.75655 |
| 19848 | Rnu2-10 | U2 small nuclear RNA 10 | -0.75478 |
| 17141 | Magea5 | melanoma antigen, family A, 5 | -0.74846 |
| 20512 | Slc1a3 | solute carrier family 1 (glial high affinity glutamate transporter), member 3 | -0.74688 |
| 67286 | Ift22 | intraflagellar transport 22 | -0.73812 |
| 67896 | Ccdc80 | coiled-coil domain containing 80 | -0.72881 |
| NM_001305678 | - | - | -0.72688 |
| 52829 | Lurap1l | leucine rich adaptor protein 1-like | -0.72108 |
| 195046 | Nlrp1a | NLR family, pyrin domain containing 1A | -0.71989 |
| 13601 | Ecm1 | extracellular matrix protein 1 | -0.71705 |
| 26457 | Slc27a1 | solute carrier family 27 (fatty acid transporter), member 1 | -0.71702 |
| 20306 | Ccl7 | chemokine (C-C motif) ligand 7 | -0.71468 |
| 102644 | Oaf | OAF homolog (Drosophila) | -0.71263 |
| 11535 | Adm | adrenomedullin | -0.70994 |
| 14178 | Fgf7 | fibroblast growth factor 7 | -0.7077 |
| 15184 | Hdac5 | histone deacetylase 5 | -0.70425 |
| 12183 | Bpgm | 2,3-bisphosphoglycerate mutase | -0.70311 |
| 14201 | Fhl3 | four and a half LIM domains 3 | -0.69913 |
| 21648 | Dynlt1b | dynein light chain Tctex-type 1B | -0.69137 |
| 68646 | Nadk2 | NAD kinase 2, mitochondrial | -0.68886 |
| 13010 | Cst3 | cystatin C | -0.68472 |
| 434866 | Gm15127 | predicted gene 15127 | -0.68467 |
| 69692 | Hddc2 | HD domain containing 2 | -0.68383 |
| 544678 | 2010015L04Rik | cilia and flagella associated protein 74 | -0.66383 |
| 68040 | Zfp593 | zinc finger protein 593 | -0.66141 |
| 14238 | Foxf2 | forkhead box F2 | -0.66124 |
| 21825 | Thbs1 | thrombospondin 1 | -0.64913 |
| 66136 | Znrd1 | zinc ribbon domain containing, 1 | -0.64797 |
| 20200 | S100a6 | S100 calcium binding protein A6 (calcyclin) | -0.62531 |
| 20750 | Spp1 | secreted phosphoprotein 1 | -0.62402 |
| 208228 | Mob3a | MOB kinase activator 3A | -0.62202 |
| 70153 | 2210016F16Rik | RIKEN cDNA 2210016F16 gene | -0.61588 |
| 20643 | Snrpe | small nuclear ribonucleoprotein E | -0.60817 |
| 100529082 | Gm11127 | predicted gene 11127 | -0.60226 |
| 234814 | Mthfsd | methenyltetrahydrofolate synthetase domain containing | -0.60032 |
| 66460 | Sys1 | SYS1 Golgi-localized integral membrane protein homolog (S. cerevisiae) | -0.59997 |
| 14470 | Rabac1 | Rab acceptor 1 (prenylated) | -0.59584 |
| NM_001305643 | - | - | -0.5942 |
| 223453 | Dap | death-associated protein | -0.58853 |
| 20198 | S100a4 | S100 calcium binding protein A4 | -0.58513 |
| 75815 | 4930470H14Rik | RIKEN cDNA 4930470H14 gene | 0.584963 |
| 229227 | 4932438A13Rik | RIKEN cDNA 4932438A13 gene | 0.588666 |
| 67579 | Cpeb4 | cytoplasmic polyadenylation element binding protein 4 | 0.589368 |
| 240427 | Setbp1 | SET binding protein 1 | 0.589492 |
| 239667 | Dip2b | DIP2 disco-interacting protein 2 homolog B (Drosophila) | 0.589963 |
| 229542 | Gatad2b | GATA zinc finger domain containing 2B | 0.590615 |
| 56531 | Ylpm1 | YLP motif containing 1 | 0.590876 |
| 209039 | Tenc1 | tensin like C1 domain-containing phosphatase | 0.591228 |
| 16599 | Klf3 | Kruppel-like factor 3 (basic) | 0.592692 |
| 239719 | Mkl2 | MKL/myocardin-like 2 | 0.593911 |
| 70683 | Utp20 | UTP20, small subunit (SSU) processome component, homolog (yeast) | 0.594019 |
| 100039934 | Gm15085 | predicted gene 15085 | 0.594435 |
| 18015 | Nf1 | neurofibromatosis 1 | 0.599201 |
| 22592 | Ercc5 | excision repair cross-complementing rodent repair deficiency, complementation group 5 | 0.599517 |
| 57258 | Xpo4 | exportin 4 | 0.601326 |
| 237615 | Ankrd52 | ankyrin repeat domain 52 | 0.601358 |
| 12353 | Car6 | carbonic anhydrase 6 | 0.601426 |
| 225115 | Svil | supervillin | 0.602072 |
| 192287 | Slc25a36 | solute carrier family 25, member 36 | 0.602665 |
| 66395 | Ahnak | AHNAK nucleoprotein (desmoyokin) | 0.602972 |
| 245622 | Fam199x | family with sequence similarity 199, X-linked | 0.604954 |
| 75415 | Arhgap12 | Rho GTPase activating protein 12 | 0.606143 |
| 242474 | Tmem245 | transmembrane protein 245 | 0.606971 |
| 71710 | Lrrcc1 | leucine rich repeat and coiled-coil domain containing 1 | 0.610882 |
| 353211 | Prune2 | prune homolog 2 (Drosophila) | 0.616863 |
| 330914 | Arhgap32 | Rho GTPase activating protein 32 | 0.616935 |
| 226562 | Prrc2c | proline-rich coiled-coil 2C | 0.61789 |
| 56807 | Scamp5 | secretory carrier membrane protein 5 | 0.619924 |
| 76580 | Mib2 | mindbomb homolog 2 (Drosophila) | 0.620519 |
| 433801 | Gm13212 | predicted gene 13212 | 0.622349 |
| 70297 | Gcc2 | GRIP and coiled-coil domain containing 2 | 0.622781 |
| 21872 | Tjp1 | tight junction protein 1 | 0.623652 |
| 18846 | Plxna3 | plexin A3 | 0.623782 |
| 16564 | Kif21a | kinesin family member 21A | 0.626944 |
| 18286 | Odf2 | outer dense fiber of sperm tails 2 | 0.62995 |
| 75785 | Klhl24 | kelch-like 24 | 0.631032 |
| 98910 | Usp6nl | USP6 N-terminal like | 0.63417 |
| 17355 | Aff1 | AF4/FMR2 family, member 1 | 0.635312 |
| 77097 | Tanc2 | tetratricopeptide repeat, ankyrin repeat and coiled-coil containing 2 | 0.63895 |
| 224650 | Anks1 | ankyrin repeat and SAM domain containing 1 | 0.639253 |
| 237886 | Slfn9 | schlafen 9 | 0.639557 |
| 80292 | Zxdc | ZXD family zinc finger C | 0.640091 |
| 230895 | Vps13d | vacuolar protein sorting 13 D (yeast) | 0.641865 |
| 207521 | Dtx4 | deltex 4 homolog (Drosophila) | 0.643521 |
| 12450 | Ccng1 | cyclin G1 | 0.644002 |
| 208846 | Daam1 | dishevelled associated activator of morphogenesis 1 | 0.64689 |
| 108075 | Ltbp4 | latent transforming growth factor beta binding protein 4 | 0.648256 |
| 241915 | Phc3 | polyhomeotic-like 3 (Drosophila) | 0.648273 |
| 233071 | Arhgap33 | Rho GTPase activating protein 33 | 0.652469 |
| 226251 | Ablim1 | actin-binding LIM protein 1 | 0.653589 |
| 320528 | Vps13c | vacuolar protein sorting 13C (yeast) | 0.654797 |
| 268980 | Strn | striatin, calmodulin binding protein | 0.654835 |
| 11898 | Ass1 | argininosuccinate synthetase 1 | 0.656149 |
| 73910 | Arhgap18 | Rho GTPase activating protein 18 | 0.658238 |
| 226525 | Rasal2 | RAS protein activator like 2 | 0.65966 |
| 94284 | Ugt1a6a | UDP glucuronosyltransferase 1 family, polypeptide A6A | 0.660396 |
| 232339 | Ankrd26 | ankyrin repeat domain 26 | 0.661655 |
| 235344 | Sik2 | salt inducible kinase 2 | 0.662965 |
| 56381 | Spen | SPEN homolog, transcriptional regulator (Drosophila) | 0.668 |
| 70012 | Cep85 | centrosomal protein 85 | 0.668572 |
| 223669 | Zfp7 | zinc finger protein 7 | 0.668794 |
| 54446 | Nfat5 | nuclear factor of activated T cells 5 | 0.669719 |
| 104836 | Cbll1 | Casitas B-lineage lymphoma-like 1 | 0.670345 |
| 20403 | Itsn2 | intersectin 2 | 0.670876 |
| 70292 | Afap1 | actin filament associated protein 1 | 0.677567 |
| 320506 | Lmbrd2 | LMBR1 domain containing 2 | 0.680217 |
| 22427 | Wrn | Werner syndrome homolog (human) | 0.683878 |
| 66573 | Dzip1 | DAZ interacting protein 1 | 0.686223 |
| 71643 | Zgrf1 | zinc finger, GRF-type containing 1 | 0.68926 |
| 277939 | C2cd3 | C2 calcium-dependent domain containing 3 | 0.692549 |
| 76464 | Casc5 | cancer susceptibility candidate 5 | 0.696908 |
| 665001 | Gm14391 | predicted gene 14391 | 0.698536 |
| 269614 | Pank4 | pantothenate kinase 4 | 0.707101 |
| 16579 | Kifap3 | kinesin-associated protein 3 | 0.707966 |
| 381022 | Kmt2d | lysine (K)-specific methyltransferase 2D | 0.709291 |
| 15258 | Hipk2 | homeodomain interacting protein kinase 2 | 0.709891 |
| 21951 | Tnks | tankyrase, TRF1-interacting ankyrin-related ADP-ribose polymerase | 0.710932 |
| 24132 | Zfp53 | zinc finger protein 53 | 0.711851 |
| 100037282 | Rsph3b | radial spoke 3B homolog (Chlamydomonas) | 0.712793 |
| 209334 | Gen1 | Gen homolog 1, endonuclease (Drosophila) | 0.713623 |
| 230848 | Zbtb40 | zinc finger and BTB domain containing 40 | 0.720122 |
| 77777 | Ulbp1 | UL16 binding protein 1 | 0.726065 |
| 665211 | Gm14326 | predicted gene 14326 | 0.728803 |
| 76967 | 2700049A03Rik | RIKEN cDNA 2700049A03 gene | 0.729548 |
| 57435 | Plin4 | perilipin 4 | 0.730013 |
| 626391 | Zfp951 | zinc finger protein 951 | 0.730942 |
| 212281 | A530054K11Rik | RIKEN cDNA A530054K11 gene | 0.731183 |
| 240476 | Zfp407 | zinc finger protein 407 | 0.731511 |
| 384309 | Trim56 | tripartite motif-containing 56 | 0.73171 |
| 100034361 | Mfap1b | microfibrillar-associated protein 1B | 0.734057 |
| 268749 | Rnf31 | ring finger protein 31 | 0.741082 |
| 433926 | Lrrc8b | leucine rich repeat containing 8 family, member B | 0.74786 |
| 242083 | Ppm1l | protein phosphatase 1 (formerly 2C)-like | 0.749511 |
| 109181 | Trip11 | thyroid hormone receptor interactor 11 | 0.750171 |
| 26920 | Cntrl | centriolin | 0.750222 |
| 20336 | Exoc4 | exocyst complex component 4 | 0.751922 |
| 26401 | Map3k1 | mitogen-activated protein kinase kinase kinase 1 | 0.758027 |
| 257633 | Acsf3 | acyl-CoA synthetase family member 3 | 0.758736 |
| 269152 | Kif26b | kinesin family member 26B | 0.763702 |
| 408022 | Primpol | primase and polymerase (DNA-directed) | 0.765794 |
| 27015 | Polk | polymerase (DNA directed), kappa | 0.771135 |
| 320404 | Itpkb | inositol 1,4,5-trisphosphate 3-kinase B | 0.772747 |
| 68916 | Cdkal1 | CDK5 regulatory subunit associated protein 1-like 1 | 0.776562 |
| 17864 | Mybl1 | myeloblastosis oncogene-like 1 | 0.791333 |
| 19714 | Rev3l | REV3-like, catalytic subunit of DNA polymerase zeta RAD54 like (S. cerevisiae) | 0.793379 |
| 13544 | Dvl3 | dishevelled 3, dsh homolog (Drosophila) | 0.796283 |
| 12190 | Brca2 | breast cancer 2 | 0.802726 |
| 71898 | Apol9b | apolipoprotein L 9b | 0.804361 |
| 59090 | Midn | midnolin | 0.807144 |
| 239122 | Setdb2 | SET domain, bifurcated 2 | 0.807355 |
| 13714 | Elk4 | ELK4, member of ETS oncogene family | 0.812172 |
| 223922 | Atf7 | activating transcription factor 7 | 0.813008 |
| 105171 | Arrdc3 | arrestin domain containing 3 | 0.813641 |
| 233552 | Gdpd5 | glycerophosphodiester phosphodiesterase domain containing 5 | 0.819062 |
| 77057 | Ston1 | stonin 1 | 0.820746 |
| 223672 | Apol9a | apolipoprotein L 9a | 0.824873 |
| 260315 | Nav3 | neuron navigator 3 | 0.828047 |
| 214150 | Ago3 | argonaute RISC catalytic subunit 3 | 0.831602 |
| 224836 | Usp49 | ubiquitin specific peptidase 49 | 0.835442 |
| 12728 | Clcn5 | chloride channel 5 | 0.840442 |
| 68817 | Ddi2 | DNA-damage inducible protein 2 | 0.844129 |
| 104027 | Synpo | synaptopodin | 0.851656 |
| 245527 | Eda2r | ectodysplasin A2 receptor | 0.860725 |
| 24127 | Xrn1 | 5'-3' exoribonuclease 1 | 0.862775 |
| 104799 | Vipas39 | VPS33B interacting protein, apical-basolateral polarity regulator, spe-39 homolog | 0.862781 |
| 11783 | Apaf1 | apoptotic peptidase activating factor 1 | 0.869712 |
| 19645 | Rb1 | retinoblastoma 1 | 0.887014 |
| 320871 | B230206H07Rik | RIKEN cDNA B230206H07 gene | 0.889235 |
| 17920 | Myo6 | myosin VI | 0.889926 |
| 667118 | Zbed6 | zinc finger, BED domain containing 6 | 0.898519 |
| 20324 | Sdpr | serum deprivation response | 0.909442 |
| 23966 | Tenm4 | teneurin transmembrane protein 4 | 0.920199 |
| 442834 | D830031N03Rik | RIKEN cDNA D830031N03 gene | 0.922379 |
| 230316 | Megf9 | multiple EGF-like-domains 9 | 0.924 |
| 333789 | N4bp2 | NEDD4 binding protein 2 | 0.924813 |
| 223650 | Eppk1 | epiplakin 1 | 0.931828 |
| 18640 | Pfkfb2 | 6-phosphofructo-2-kinase/fructose-2,6-biphosphatase 2 | 0.934782 |
| 626534 | Gm6682 | tubulin, alpha 1C pseudogene | 0.969015 |
| 19712 | Rest | RE1-silencing transcription factor | 0.972794 |
| 12704 | Cit | citron | 0.974976 |
| 382090 | Cep162 | centrosomal protein 162 | 0.992928 |
| 238386 | Btbd7 | BTB (POZ) domain containing 7 | 1.050245 |
| 241520 | Fam171b | family with sequence similarity 171, member B | 1.05322 |
| 16554 | Kif13b | kinesin family member 13B | 1.059589 |
| 75869 | Arl5b | ADP-ribosylation factor-like 5B | 1.061401 |
| 170936 | Zfp369 | zinc finger protein 369 | 1.144624 |
| 235047 | Zfp809 | zinc finger protein 809 | 1.146916 |
| 66808 | 9030624G23Rik | RIKEN cDNA 9030624G23 gene | 1.190622 |
| 12503 | Cd247 | CD247 antigen | 1.194647 |
| 17101 | Lyst | lysosomal trafficking regulator | 1.203634 |
| 16589 | Uhmk1 | U2AF homology motif (UHM) kinase 1 | 1.214866 |
| 14536 | Nr6a1 | nuclear receptor subfamily 6, group A, member 1 | 1.222392 |
| 11496 | Adam22 | a disintegrin and metallopeptidase domain 22 | 1.273648 |
| 100503240 | Trpc5os | transient receptor potential cation channel, subfamily C, member 5, opposite strand | 1.315355 |
| 170829 | Tram2 | translocating chain-associating membrane protein 2 | 1.325812 |
| 11670 | Aldh3a1 | aldehyde dehydrogenase family 3, subfamily A1 | 1.346226 |
| 11567 | Avil | advillin | 1.378006 |
| 664987 | Gm14393 | predicted gene 14393 | 1.382423 |
| 407812 | Zfp941 | zinc finger protein 941 | 1.43105 |
| 219150 | Hmbox1 | homeobox containing 1 | 1.459432 |
| 100216455 | Gm14124 | predicted gene 14124 | 1.463247 |
| 23886 | Gdf15 | growth differentiation factor 15 | 1.469646 |
| 667666 | Zfp600 | zinc finger protein 600 | 1.542276 |
| 319586 | Celf5 | CUGBP, Elav-like family member 5 | 1.552541 |
| 71111 | Gpr39 | G protein-coupled receptor 39 | 1.592576 |
| 100041515 | Gm3383 | predicted gene 3383 | 1.660911 |
| 268903 | Nrip1 | nuclear receptor interacting protein 1 | 1.66432 |
| 14349 | Fv1 | Friend virus susceptibility 1 | 1.782562 |
| 434197 | Fam169b | family with sequence similarity 169, member B | 1.830075 |
| 665005 | Gm7444 | ring finger protein 19A pseudogene | 1.913288 |
| 66689 | Klhl28 | kelch-like 28 | 2.148099 |
| 631304 | Cyp4f40 | cytochrome P450, family 4, subfamily f, polypeptide 40 | 2.231564 |
| 20148 | Dhrs3 | dehydrogenase/reductase (SDR family) member 3 | 2.465267 |
| 246727 | Oas3 | 2'-5' oligoadenylate synthetase 3 | 2.887525 |
| 668030 | Gm14432 | predicted gene 14432 | 2.892391 |
| 229898 | Gbp5 | guanylate binding protein 5 | 3.025535 |
| 11695 | Alx4 | aristaless-like homeobox 4 | 3.36257 |
| 100038725 | Cep85l | centrosomal protein 85-like | 3.375039 |
| 100504249 | Gm15350 | predicted gene 15350 | 3.477617 |
| 69066 | 1810010H24Rik | RIKEN cDNA 1810010H24 gene | 3.566815 |
| 26888 | Clec4a2 | C-type lectin domain family 4, member a2 | 9.569856 |
| 226075 | Glis3 | GLIS family zinc finger 3 | 9.98014 |
| 102238433 | Jmjd7-pla2g4b | predicted gene, 28042 | 10.17991 |
| 71607 | Snx20 | sorting nexin 20 | 10.22882 |
| NR_131218 | - | - | 10.47168 |
| NM_001305668 | - | - | 10.72281 |
| 545651 | Gm13278 | predicted gene 13278 | 10.98726 |
| 69750 | 2410021H03Rik | RIKEN cDNA 2410021H03 gene | 11.49685 |
| NM_001305843 | - | - | 11.55075 |
| NM_001305631 | - | - | 11.79766 |
| 319172 | Hist1h2ab | histone cluster 1, H2ab | 11.94764 |
| 21822 | Tgtp1 | T cell specific GTPase 1 | 12.21371 |
| 319161 | Hist1h4n | histone cluster 1, H4n | 12.93664 |
| 627914 | Gm14430 | predicted gene 14430 | 13.26004 |
| 319170 | Hist1h2an | histone cluster 1, H2an | 14.64723 |

**Supplementary Table S3** The list of genes which were differentially expressed in glutamate plus SA extract treatment compared with glutamate treatment HT22 cells.

| **geneID** | **Symbol** | **Description** | **Expr Log Ratio** |
| --- | --- | --- | --- |
| 319191 | Hist1h2ai | histone cluster 1, H2ai | -14.6033 |
| 654432 | Gm7334 | B-cell translocation gene 3 pseudogene | -13.0293 |
| 319181 | Hist1h2bg | histone cluster 1, H2bg | -12.6348 |
| NR_131219 | - | - | -12.1196 |
| NM_001305819 | - | - | -11.9403 |
| 100039116 | Mup14 | major urinary protein 14 | -11.5459 |
| 100039206 | Mup17 | major urinary protein 17 | -11.3106 |
| 100039054 | Mup12 | major urinary protein 12 | -11.2819 |
| 100042295 | Gm3776 | predicted gene 3776 | -11.2703 |
| 100039240 | Gm10058 | predicted gene 10058 | -11.1861 |
| 100042149 | Gm3696 | predicted gene 3696 | -10.8533 |
| NR_131225 | - | - | -10.2761 |
| 140795 | P2ry14 | purinergic receptor P2Y, G-protein coupled, 14 | -10.2167 |
| 100041515 | Gm3383 | predicted gene 3383 | -10.1163 |
| 100568458 | Raver1-fdx1l | Raver1-Fdx1l readthrough | -10.0768 |
| NM_001305675 | - | - | -9.90689 |
| 12895 | Cpt1b | carnitine palmitoyltransferase 1b, muscle | -9.71425 |
| 320078 | Olfml2b | olfactomedin-like 2B | -9.07682 |
| 271849 | Shc4 | SHC (Src homology 2 domain containing) family, member 4 | -8.60733 |
| 16516 | Kcnj15 | potassium inwardly-rectifying channel, subfamily J, member 15 | -8.40939 |
| 218865 | Chdh | choline dehydrogenase | -8.27612 |
| 22172 | Tyms-ps | thymidylate synthase, pseudogene | -4.82443 |
| NM_001305844 | - | - | -2.85457 |
| 76743 | Gje1 | gap junction protein, epsilon 1 | -2.63126 |
| 56485 | Slc2a5 | solute carrier family 2 (facilitated glucose transporter), member 5 | -2.42146 |
| 26563 | Ror1 | receptor tyrosine kinase-like orphan receptor 1 | -2.39993 |
| 28105 | Trim36 | tripartite motif-containing 36 | -2.37504 |
| 53901 | Rcan2 | regulator of calcineurin 2 | -2.31079 |
| 77462 | Tmem116 | transmembrane protein 116 | -1.90913 |
| 69576 | Smco1 | single-pass membrane protein with coiled-coil domains 1 | -1.87584 |
| 21923 | Tnc | tenascin C | -1.7409 |
| 19225 | Ptgs2 | prostaglandin-endoperoxide synthase 2 | -1.7216 |
| 14816 | Grm1 | glutamate receptor, metabotropic 1 | -1.65208 |
| 211429 | Pla2g4b | phospholipase A2, group IVB (cytosolic) | -1.6495 |
| 23886 | Gdf15 | growth differentiation factor 15 | -1.63198 |
| 545276 | Gal3st3 | galactose-3-O-sulfotransferase 3 | -1.61298 |
| 20269 | Scn3a | sodium channel, voltage-gated, type III, alpha | -1.60334 |
| 17082 | Il1rl1 | interleukin 1 receptor-like 1 | -1.58236 |
| 16449 | Jag1 | jagged 1 | -1.53051 |
| 216792 | Iba57 | IBA57, iron-sulfur cluster assembly homolog (S. cerevisiae) | -1.49855 |
| 64817 | Svep1 | sushi, von Willebrand factor type A, EGF and pentraxin domain containing 1 | -1.40336 |
| 269614 | Pank4 | pantothenate kinase 4 | -1.38793 |
| 100038752 | Gm10825 | predicted gene 10825 | -1.33065 |
| 12835 | Col6a3 | collagen, type VI, alpha 3 | -1.32977 |
| 121021 | Cspg4 | chondroitin sulfate proteoglycan 4 | -1.30521 |
| 100039890 | Gm15093 | predicted gene 15093 | -1.29445 |
| 14165 | Fgf10 | fibroblast growth factor 10 | -1.23733 |
| 67531 | 5730408K05Rik | RIKEN cDNA 5730408K05 gene | -1.21957 |
| 244668 | Sipa1l2 | signal-induced proliferation-associated 1 like 2 | -1.18587 |
| 11806 | Apoa1 | apolipoprotein A-I | -1.17716 |
| 399558 | Flrt2 | fibronectin leucine rich transmembrane protein 2 | -1.17306 |
| 64113 | Moap1 | modulator of apoptosis 1 | -1.16866 |
| 70005 | Znf41-ps | ZNF41, pseudogene | -1.16463 |
| 14066 | F3 | coagulation factor III | -1.15835 |
| 16492 | Kcna4 | potassium voltage-gated channel, shaker-related subfamily, member 4 | -1.14066 |
| 21788 | Tfpi | tissue factor pathway inhibitor | -1.14037 |
| 17841 | Mup2 | major urinary protein 2 | -1.11459 |
| 229595 | Adamtsl4 | ADAMTS-like 4 | -1.09464 |
| 19819 | Rnaseh1 | ribonuclease H1 | -1.08691 |
| 18574 | Pde1b | phosphodiesterase 1B, Ca2+-calmodulin dependent | -1.08406 |
| 76080 | Ttpal | tocopherol (alpha) transfer protein-like | -1.07929 |
| 73106 | Prss57 | protease, serine 57 | -1.06784 |
| 12826 | Col4a1 | collagen, type IV, alpha 1 | -1.05403 |
| 21825 | Thbs1 | thrombospondin 1 | -1.05343 |
| 19288 | Ptx3 | pentraxin related gene | -1.05271 |
| 208501 | 1810043H04Rik | RIKEN cDNA 1810043H04 gene | -1.04173 |
| 14314 | Fstl1 | follistatin-like 1 | -1.03747 |
| 14806 | Grik2 | glutamate receptor, ionotropic, kainate 2 (beta 2) | -1.03317 |
| 17158 | Man2a1 | mannosidase 2, alpha 1 | -1.02616 |
| 13003 | Vcan | versican | -1.02017 |
| 21803 | Tgfb1 | transforming growth factor, beta 1 | -1.01681 |
| 381290 | Atp2b4 | ATPase, Ca++ transporting, plasma membrane 4 | -1.01668 |
| 18811 | Prl2c2 | prolactin family 2, subfamily c, member 2 | -1.01318 |
| 102465886 | Mir8094 | microRNA mir-8094 | -1.00375 |
| 71306 | Mfap3l | microfibrillar-associated protein 3-like | -1 |
| 78512 | 3300005D01Rik | RIKEN cDNA 3300005D01 gene | -0.99077 |
| 67014 | Mina | myc induced nuclear antigen | -0.98697 |
| 17155 | Man1a | mannosidase 1, alpha | -0.97211 |
| 16948 | Lox | lysyl oxidase | -0.96598 |
| 18612 | Etv4 | ets variant 4 | -0.96452 |
| 102423 | Hinfp | histone H4 transcription factor | -0.96297 |
| 232975 | Atp1a3 | ATPase, Na+/K+ transporting, alpha 3 polypeptide | -0.96178 |
| 15365 | Hmga2-ps1 | high mobility group AT-hook 2, pseudogene 1 | -0.9409 |
| 16323 | Inhba | inhibin beta-A | -0.92578 |
| 13340 | Slc29a2 | solute carrier family 29 (nucleoside transporters), member 2 | -0.92373 |
| 246293 | Klhl8 | kelch-like 8 | -0.91623 |
| 381677 | Vgf | VGF nerve growth factor inducible | -0.91112 |
| 16007 | Cyr61 | cysteine rich protein 61 | -0.91035 |
| 19848 | Rnu2-10 | U2 small nuclear RNA 10 | -0.90396 |
| 67892 | Coa6 | cytochrome c oxidase assembly factor 6 | -0.90079 |
| 68875 | Tmcc2 | transmembrane and coiled-coil domains 2 | -0.90071 |
| 11504 | Adamts1 | a disintegrin-like and metallopeptidase (reprolysin type) with thrombospondin type 1 motif, 1 | -0.89985 |
| 14118 | Fbn1 | fibrillin 1 | -0.89853 |
| 14590 | Ggh | gamma-glutamyl hydrolase | -0.89583 |
| 12827 | Col4a2 | collagen, type IV, alpha 2 | -0.8893 |
| NM_001305643 | - | - | -0.88736 |
| 22782 | Slc30a1 | solute carrier family 30 (zinc transporter), member 1 | -0.88368 |
| 20348 | Sema3c | sema domain, immunoglobulin domain (Ig), short basic domain, secreted, (semaphorin) 3C | -0.88296 |
| 319493 | A430078G23Rik | RIKEN cDNA A430078G23 gene | -0.86488 |
| 68628 | Fbxw9 | F-box and WD-40 domain protein 9 | -0.85528 |
| 217344 | Rhbdf2 | rhomboid 5 homolog 2 (Drosophila) | -0.8521 |
| 72265 | Tram1 | translocating chain-associating membrane protein 1 | -0.85091 |
| 100529082 | Gm11127 | predicted gene 11127 | -0.85004 |
| 13601 | Ecm1 | extracellular matrix protein 1 | -0.84894 |
| 18787 | Serpine1 | serine (or cysteine) peptidase inhibitor, clade E, member 1 | -0.84697 |
| 68040 | Zfp593 | zinc finger protein 593 | -0.84567 |
| 83554 | Fstl3 | follistatin-like 3 | -0.83878 |
| 12497 | Entpd6 | ectonucleoside triphosphate diphosphohydrolase 6 | -0.83773 |
| 17112 | Tm4sf1 | transmembrane 4 superfamily member 1 | -0.83764 |
| 14107 | Fat1 | FAT tumor suppressor homolog 1 (Drosophila) | -0.8371 |
| 217887 | BC022687 | cDNA sequence BC022687 | -0.83026 |
| 12490 | Cd34 | CD34 antigen | -0.82241 |
| 100040617 | Gm12603 | predicted gene 12603 | -0.81578 |
| 50794 | Klf13 | Kruppel-like factor 13 | -0.80992 |
| 235505 | Cd109 | CD109 antigen | -0.79944 |
| 234857 | Spire2 | spire homolog 2 (Drosophila) | -0.79905 |
| 109305 | Orai1 | ORAI calcium release-activated calcium modulator 1 | -0.79821 |
| 15368 | Hmox1 | heme oxygenase (decycling) 1 | -0.78916 |
| 20509 | Slc19a1 | solute carrier family 19 (folate transporter), member 1 | -0.78533 |
| 19124 | Procr | protein C receptor, endothelial | -0.78508 |
| 100415914 | Gm12505 | predicted gene 12505 | -0.77366 |
| 15586 | Hyal1 | hyaluronoglucosaminidase 1 | -0.77259 |
| 14238 | Foxf2 | forkhead box F2 | -0.7705 |
| 228859 | Fitm2 | fat storage-inducing transmembrane protein 2 | -0.76847 |
| 338364 | Trim65 | tripartite motif-containing 65 | -0.76796 |
| 234854 | Cdk10 | cyclin-dependent kinase 10 | -0.76647 |
| 279653 | Pcdh19 | protocadherin 19 | -0.76219 |
| 68861 | 1190002N15Rik | RIKEN cDNA 1190002N15 gene | -0.75713 |
| 66435 | Uggt2 | UDP-glucose glycoprotein glucosyltransferase 2 | -0.74582 |
| 20512 | Slc1a3 | solute carrier family 1 (glial high affinity glutamate transporter), member 3 | -0.74086 |
| 100039864 | Snhg12 | small nucleolar RNA host gene 12 | -0.73665 |
| 22041 | Trf | transferrin | -0.73323 |
| 14178 | Fgf7 | fibroblast growth factor 7 | -0.73155 |
| 94352 | Loxl2 | lysyl oxidase-like 2 | -0.73039 |
| 18514 | Pbx1 | pre B cell leukemia homeobox 1 | -0.72706 |
| 14115 | Fbln2 | fibulin 2 | -0.7236 |
| 14824 | Grn | granulin | -0.71285 |
| 230861 | Eif4g3 | eukaryotic translation initiation factor 4 gamma, 3 | -0.71128 |
| 20296 | Ccl2 | chemokine (C-C motif) ligand 2 | -0.70798 |
| 268977 | Ltbp1 | latent transforming growth factor beta binding protein 1 | -0.70157 |
| 666184 | Gm15080 | predicted gene 15080 | -0.70059 |
| 504193 | Npcd | neuronal pentraxin chromo domain | -0.69788 |
| 16777 | Lamb1 | laminin B1 | -0.69348 |
| 14283 | Fosl1 | fos-like antigen 1 | -0.69335 |
| 18129 | Notch2 | notch 2 | -0.69278 |
| 100042165 | BC005561 | cDNA sequence BC005561 | -0.69135 |
| 69675 | Pxdn | peroxidasin homolog (Drosophila) | -0.68356 |
| 239027 | Arhgap22 | Rho GTPase activating protein 22 | -0.68286 |
| 58235 | Pvrl1 | poliovirus receptor-related 1 | -0.68038 |
| 384783 | Irs2 | insulin receptor substrate 2 | -0.67904 |
| 207740 | Ubald1 | UBA-like domain containing 1 | -0.6788 |
| 16779 | Lamb2 | laminin, beta 2 | -0.67149 |
| 230752 | Eva1b | eva-1 homolog B (C. elegans) | -0.66963 |
| 246154 | Vasn | vasorin | -0.66675 |
| 17967 | Ncam1 | neural cell adhesion molecule 1 | -0.66454 |
| 26457 | Slc27a1 | solute carrier family 27 (fatty acid transporter), member 1 | -0.66382 |
| 320951 | Pisd | phosphatidylserine decarboxylase | -0.66284 |
| 226122 | Ubtd1 | ubiquitin domain containing 1 | -0.65984 |
| 52118 | Pvr | poliovirus receptor | -0.6582 |
| 64075 | Smoc1 | SPARC related modular calcium binding 1 | -0.65532 |
| 665155 | Srp54b | signal recognition particle 54B | -0.64257 |
| 72599 | Pdia5 | protein disulfide isomerase associated 5 | -0.64076 |
| 16956 | Lpl | lipoprotein lipase | -0.64059 |
| 12831 | Col5a1 | collagen, type V, alpha 1 | -0.63545 |
| 14860 | Gsta4 | glutathione S-transferase, alpha 4 | -0.63326 |
| 14268 | Fn1 | fibronectin 1 | -0.63124 |
| 67896 | Ccdc80 | coiled-coil domain containing 80 | -0.62756 |
| 15530 | Hspg2 | perlecan (heparan sulfate proteoglycan 2) | -0.62721 |
| 23794 | Adamts5 | a disintegrin-like and metallopeptidase (reprolysin type) with thrombospondin type 1 motif, 5 (aggrecanase-2) | -0.6249 |
| 27419 | Naglu | alpha-N-acetylglucosaminidase (Sanfilippo disease IIIB) | -0.62385 |
| 17387 | Mmp14 | matrix metallopeptidase 14 (membrane-inserted) | -0.62215 |
| 18301 | Fxyd5 | FXYD domain-containing ion transport regulator 5 | -0.61894 |
| 12832 | Col5a2 | collagen, type V, alpha 2 | -0.61567 |
| 15184 | Hdac5 | histone deacetylase 5 | -0.61517 |
| 66190 | Acer3 | alkaline ceramidase 3 | -0.61339 |
| 12795 | Plk3 | polo-like kinase 3 | -0.6125 |
| 269941 | Chsy1 | chondroitin sulfate synthase 1 | -0.61017 |
| 226519 | Lamc1 | laminin, gamma 1 | -0.60761 |
| 72544 | Exosc6 | exosome component 6 | -0.60392 |
| 12727 | Clcn4-2 | chloride channel 4-2 | -0.60293 |
| 14299 | Ncs1 | neuronal calcium sensor 1 | -0.60283 |
| 83768 | Dpp7 | dipeptidylpeptidase 7 | -0.60253 |
| 14466 | Gba | glucosidase, beta, acid | -0.59853 |
| 320878 | Mical2 | microtubule associated monooxygenase, calponin and LIM domain containing 2 | -0.59793 |
| 319171 | Hist1h2ap | histone cluster 1, H2ap | -0.59728 |
| 16402 | Itga5 | integrin alpha 5 (fibronectin receptor alpha) | -0.596 |
| 12505 | Cd44 | CD44 antigen | -0.59553 |
| 11605 | Gla | galactosidase, alpha | -0.59409 |
| 20652 | Soat1 | sterol O-acyltransferase 1 | -0.59262 |
| NR_131212 | - | - | -0.59123 |
| 12111 | Bgn | biglycan | -0.5901 |
| 19156 | Psap | prosaposin | -0.5863 |
| 17181 | Matn2 | matrilin 2 | -0.58583 |
| 12390 | Cav2 | caveolin 2 | -0.58562 |
| 23849 | Klf6 | Kruppel-like factor 6 | -0.58526 |
| 13822 | Epb4.1l2 | erythrocyte protein band 4.1-like 2 | 0.587029 |
| 338467 | Morc3 | microrchidia 3 | 0.587924 |
| 66573 | Dzip1 | DAZ interacting protein 1 | 0.589044 |
| 12190 | Brca2 | breast cancer 2 | 0.589269 |
| 230673 | Ipo13 | importin 13 | 0.590024 |
| 30941 | Usp21 | ubiquitin specific peptidase 21 | 0.592661 |
| 66522 | Pgpep1 | pyroglutamyl-peptidase I | 0.593588 |
| 56807 | Scamp5 | secretory carrier membrane protein 5 | 0.59441 |
| 103677 | Smg6 | Smg-6 homolog, nonsense mediated mRNA decay factor (C. elegans) | 0.597979 |
| 14645 | Glul | glutamate-ammonia ligase (glutamine synthetase) | 0.599556 |
| 229841 | Cenpe | centromere protein E | 0.601252 |
| 101565 | Ccp110 | centriolar coiled coil protein 110 | 0.603433 |
| 23936 | Lynx1 | Ly6/neurotoxin 1 | 0.603974 |
| 244895 | Peak1 | pseudopodium-enriched atypical kinase 1 | 0.604015 |
| 16782 | Lamc2 | laminin, gamma 2 | 0.604567 |
| 11907 | Ate1 | arginyltransferase 1 | 0.604591 |
| 108705 | Pttg1ip | pituitary tumor-transforming 1 interacting protein | 0.606303 |
| 73379 | Dcbld2 | discoidin, CUB and LCCL domain containing 2 | 0.6069 |
| 111173 | Erc1 | ELKS/RAB6-interacting/CAST family member 1 | 0.607497 |
| 72469 | Plcd3 | phospholipase C, delta 3 | 0.611169 |
| 22709 | Zfp51 | zinc finger protein 51 | 0.612014 |
| 70028 | Dopey2 | dopey family member 2 | 0.612484 |
| 20403 | Itsn2 | intersectin 2 | 0.612609 |
| 12632 | Cfl2 | cofilin 2, muscle | 0.613127 |
| 66049 | Rogdi | rogdi homolog (Drosophila) | 0.613237 |
| 74879 | 4930461G14Rik | RIKEN cDNA 4930461G14 gene | 0.613488 |
| 216869 | Arrb2 | arrestin, beta 2 | 0.614157 |
| 16010 | Igfbp4 | insulin-like growth factor binding protein 4 | 0.616646 |
| 103841 | Cuedc1 | CUE domain containing 1 | 0.617429 |
| 18708 | Pik3r1 | phosphatidylinositol 3-kinase, regulatory subunit, polypeptide 1 (p85 alpha) | 0.618129 |
| 109263 | Rlf | rearranged L-myc fusion sequence | 0.618604 |
| 66540 | Fam107b | family with sequence similarity 107, member B | 0.618946 |
| 109181 | Trip11 | thyroid hormone receptor interactor 11 | 0.619552 |
| 109689 | Arrb1 | arrestin, beta 1 | 0.620096 |
| 12442 | Ccnb2 | cyclin B2 | 0.620449 |
| 76901 | Jade2 | jade family PHD finger 2 | 0.621183 |
| 75646 | Rai14 | retinoic acid induced 14 | 0.623898 |
| 207521 | Dtx4 | deltex 4 homolog (Drosophila) | 0.62517 |
| 269338 | Vps39 | vacuolar protein sorting 39 (yeast) | 0.636967 |
| 76464 | Casc5 | cancer susceptibility candidate 5 | 0.63806 |
| 21678 | Tead3 | TEA domain family member 3 | 0.640053 |
| 22344 | Vezf1 | vascular endothelial zinc finger 1 | 0.640336 |
| 67246 | 2810474O19Rik | RIKEN cDNA 2810474O19 gene | 0.640524 |
| 238386 | Btbd7 | BTB (POZ) domain containing 7 | 0.644519 |
| 233103 | 4931406P16Rik | RIKEN cDNA 4931406P16 gene | 0.645696 |
| 209334 | Gen1 | Gen homolog 1, endonuclease (Drosophila) | 0.64694 |
| 22003 | Tpm1 | tropomyosin 1, alpha | 0.64834 |
| 215690 | Nav1 | neuron navigator 1 | 0.651033 |
| 628308 | Gm14420 | predicted gene 14420 | 0.653962 |
| 229543 | Ints3 | integrator complex subunit 3 | 0.65843 |
| 17864 | Mybl1 | myeloblastosis oncogene-like 1 | 0.658603 |
| 76954 | St5 | suppression of tumorigenicity 5 | 0.664365 |
| 223922 | Atf7 | activating transcription factor 7 | 0.668892 |
| 66175 | Mustn1 | musculoskeletal, embryonic nuclear protein 1 | 0.670347 |
| 68617 | Mtcl1 | microtubule crosslinking factor 1 | 0.67184 |
| 214239 | A430105I19Rik | RIKEN cDNA A430105I19 gene | 0.674703 |
| 230073 | Ddx58 | DEAD (Asp-Glu-Ala-Asp) box polypeptide 58 | 0.676637 |
| 77976 | Nuak1 | NUAK family, SNF1-like kinase, 1 | 0.677512 |
| 100039706 | Gm2381 | zinc finger protein 936 pseudogene | 0.678846 |
| 71361 | Aifm2 | apoptosis-inducing factor, mitochondrion-associated 2 | 0.680133 |
| 21848 | Trim24 | tripartite motif-containing 24 | 0.680188 |
| 246710 | Rhobtb2 | Rho-related BTB domain containing 2 | 0.683709 |
| 22720 | Zfp62 | zinc finger protein 62 | 0.684266 |
| 192657 | Ell2 | elongation factor RNA polymerase II 2 | 0.684655 |
| 210789 | Tbc1d4 | TBC1 domain family, member 4 | 0.686185 |
| 11677 | Akr1b3 | aldo-keto reductase family 1, member B3 (aldose reductase) | 0.687444 |
| 20454 | St3gal5 | ST3 beta-galactoside alpha-2,3-sialyltransferase 5 | 0.689751 |
| 99470 | Magi3 | membrane associated guanylate kinase, WW and PDZ domain containing 3 | 0.692513 |
| 209039 | Tenc1 | tensin like C1 domain-containing phosphatase | 0.693508 |
| 77578 | Bcl9 | B cell CLL/lymphoma 9 | 0.694048 |
| 17454 | Mov10 | Moloney leukemia virus 10 | 0.694886 |
| 18105 | Nqo2 | NAD(P)H dehydrogenase, quinone 2 | 0.696147 |
| 51793 | Ddah2 | dimethylarginine dimethylaminohydrolase 2 | 0.696248 |
| 239719 | Mkl2 | MKL/myocardin-like 2 | 0.701919 |
| 108099 | Prkag2 | protein kinase, AMP-activated, gamma 2 non-catalytic subunit | 0.701955 |
| 240476 | Zfp407 | zinc finger protein 407 | 0.709485 |
| 218613 | Mier3 | mesoderm induction early response 1, family member 3 | 0.71018 |
| 68303 | Fam114a1 | family with sequence similarity 114, member A1 | 0.714386 |
| 68259 | Ift80 | intraflagellar transport 80 | 0.715631 |
| 71839 | Osgin1 | oxidative stress induced growth inhibitor 1 | 0.718786 |
| 98845 | Eps8l2 | EPS8-like 2 | 0.727989 |
| 433801 | Gm13212 | predicted gene 13212 | 0.730755 |
| 19714 | Rev3l | REV3-like, catalytic subunit of DNA polymerase zeta RAD54 like (S. cerevisiae) | 0.730757 |
| 70207 | Taco1 | translational activator of mitochondrially encoded cytochrome c oxidase I | 0.730905 |
| 21872 | Tjp1 | tight junction protein 1 | 0.732384 |
| 14853 | Gspt2 | G1 to S phase transition 2 | 0.738826 |
| 20336 | Exoc4 | exocyst complex component 4 | 0.745038 |
| 16362 | Irf1 | interferon regulatory factor 1 | 0.746004 |
| 75723 | Amotl1 | angiomotin-like 1 | 0.747535 |
| 64297 | Gprc5b | G protein-coupled receptor, family C, group 5, member B | 0.753993 |
| 71544 | Arhgap42 | Rho GTPase activating protein 42 | 0.756976 |
| 18604 | Pdk2 | pyruvate dehydrogenase kinase, isoenzyme 2 | 0.76356 |
| 227154 | Stradb | STE20-related kinase adaptor beta | 0.767305 |
| 232431 | Gprc5a | G protein-coupled receptor, family C, group 5, member A | 0.77333 |
| 320207 | Pik3r5 | phosphoinositide-3-kinase, regulatory subunit 5, p101 | 0.773724 |
| 219103 | Cenpj | centromere protein J | 0.773831 |
| 17101 | Lyst | lysosomal trafficking regulator | 0.776328 |
| 235344 | Sik2 | salt inducible kinase 2 | 0.782608 |
| 56357 | Ivd | isovaleryl coenzyme A dehydrogenase | 0.789198 |
| 17190 | Mbd1 | methyl-CpG binding domain protein 1 | 0.792684 |
| 224836 | Usp49 | ubiquitin specific peptidase 49 | 0.79787 |
| 11783 | Apaf1 | apoptotic peptidase activating factor 1 | 0.799872 |
| 232227 | Iqsec1 | IQ motif and Sec7 domain 1 | 0.801713 |
| 77057 | Ston1 | stonin 1 | 0.811117 |
| 22698 | Zfp39 | zinc finger protein 39 | 0.81139 |
| 56791 | Ube2l6 | ubiquitin-conjugating enzyme E2L 6 | 0.817456 |
| 94242 | Tinagl1 | tubulointerstitial nephritis antigen-like 1 | 0.817797 |
| 21817 | Tgm2 | transglutaminase 2, C polypeptide | 0.821663 |
| 17127 | Smad3 | SMAD family member 3 | 0.824957 |
| 11826 | Aqp1 | aquaporin 1 | 0.825722 |
| 226251 | Ablim1 | actin-binding LIM protein 1 | 0.826255 |
| 449000 | Zfp960 | zinc finger protein 960 | 0.828904 |
| 17347 | Mknk2 | MAP kinase-interacting serine/threonine kinase 2 | 0.832008 |
| 68178 | Cgnl1 | cingulin-like 1 | 0.836629 |
| 80285 | Parp9 | poly (ADP-ribose) polymerase family, member 9 | 0.838938 |
| 231633 | Tmem119 | transmembrane protein 119 | 0.856383 |
| 12778 | Ackr3 | atypical chemokine receptor 3 | 0.869016 |
| 230895 | Vps13d | vacuolar protein sorting 13 D (yeast) | 0.869939 |
| 59090 | Midn | midnolin | 0.873799 |
| 17175 | Masp2 | mannan-binding lectin serine peptidase 2 | 0.882527 |
| 59038 | Pxmp4 | peroxisomal membrane protein 4 | 0.885375 |
| 16012 | Igfbp6 | insulin-like growth factor binding protein 6 | 0.889226 |
| 382090 | Cep162 | centrosomal protein 162 | 0.895796 |
| 71918 | Zcchc24 | zinc finger, CCHC domain containing 24 | 0.900884 |
| 319638 | Nt5dc1 | 5'-nucleotidase domain containing 1 | 0.901436 |
| 70083 | Metrn | meteorin, glial cell differentiation regulator | 0.903223 |
| 21821 | Ift88 | intraflagellar transport 88 | 0.903264 |
| 11567 | Avil | advillin | 0.906671 |
| 16579 | Kifap3 | kinesin-associated protein 3 | 0.915002 |
| 67573 | Loxl4 | lysyl oxidase-like 4 | 0.918025 |
| 71924 | Tube1 | epsilon-tubulin 1 | 0.93459 |
| 18591 | Pdgfb | platelet derived growth factor, B polypeptide | 0.938161 |
| 14756 | Gpld1 | glycosylphosphatidylinositol specific phospholipase D1 | 0.942179 |
| 54446 | Nfat5 | nuclear factor of activated T cells 5 | 0.942809 |
| 626534 | Gm6682 | tubulin, alpha 1C pseudogene | 0.94334 |
| 13821 | Epb4.1l1 | erythrocyte protein band 4.1-like 1 | 0.944174 |
| 333789 | N4bp2 | NEDD4 binding protein 2 | 0.954196 |
| 13855 | Epn2 | epsin 2 | 0.964667 |
| 18984 | Por | P450 (cytochrome) oxidoreductase | 0.972317 |
| 58207 | Slc43a3 | solute carrier family 43, member 3 | 0.973666 |
| 56047 | Msln | mesothelin | 0.977369 |
| 19411 | Rarg | retinoic acid receptor, gamma | 0.977751 |
| 18003 | Nedd9 | neural precursor cell expressed, developmentally down-regulated gene 9 | 0.980907 |
| 215653 | Rassf2 | Ras association (RalGDS/AF-6) domain family member 2 | 0.981504 |
| 231570 | A830010M20Rik | RIKEN cDNA A830010M20 gene | 0.982078 |
| 73683 | Atg16l2 | autophagy related 16-like 2 (S. cerevisiae) | 0.984233 |
| 237775 | Zfp867 | zinc finger protein 867 | 0.984403 |
| 216377 | C230029M16 | uncharacterized protein C230029M16 | 0.984504 |
| 18557 | Cdk18 | cyclin-dependent kinase 18 | 0.986737 |
| 110253 | Triobp | TRIO and F-actin binding protein | 0.98737 |
| 626009 | Gm6644 | Akr1b3 pseudogene | 0.989494 |
| 66610 | Abi3 | ABI gene family, member 3 | 0.989529 |
| 12337 | Capn5 | calpain 5 | 0.99035 |
| 65112 | Pmepa1 | prostate transmembrane protein, androgen induced 1 | 0.99123 |
| 52850 | Sgsm1 | small G protein signaling modulator 1 | 0.994782 |
| 73910 | Arhgap18 | Rho GTPase activating protein 18 | 0.99728 |
| 14469 | Gbp2 | guanylate binding protein 2 | 1.009298 |
| 66808 | 9030624G23Rik | RIKEN cDNA 9030624G23 gene | 1.012881 |
| 16589 | Uhmk1 | U2AF homology motif (UHM) kinase 1 | 1.014261 |
| 319870 | 9330136K24Rik | RIKEN cDNA 9330136K24 gene | 1.014315 |
| 21938 | Tnfrsf1b | tumor necrosis factor receptor superfamily, member 1b | 1.052467 |
| 195531 | Gm13152 | predicted gene 13152 | 1.05852 |
| 65963 | Tmem176b | transmembrane protein 176B | 1.062102 |
| 11816 | Apoe | apolipoprotein E | 1.074962 |
| 100039934 | Gm15085 | predicted gene 15085 | 1.083096 |
| 17691 | Sik1 | salt inducible kinase 1 | 1.098362 |
| 319446 | Dpep2 | dipeptidase 2 | 1.107875 |
| 20688 | Sp4 | trans-acting transcription factor 4 | 1.131245 |
| 242669 | Adc | antizyme inhibitor 2 | 1.140178 |
| 74202 | Fblim1 | filamin binding LIM protein 1 | 1.15389 |
| 76088 | Dock8 | dedicator of cytokinesis 8 | 1.161303 |
| 66058 | Tmem176a | transmembrane protein 176A | 1.168669 |
| 13609 | S1pr1 | sphingosine-1-phosphate receptor 1 | 1.170491 |
| 320871 | B230206H07Rik | RIKEN cDNA B230206H07 gene | 1.172514 |
| 12363 | Casp4 | caspase 4, apoptosis-related cysteine peptidase | 1.179324 |
| 432442 | Akap7 | A kinase (PRKA) anchor protein 7 | 1.185692 |
| 76894 | Mettl15 | methyltransferase like 15 | 1.195551 |
| 623781 | Gm14137 | predicted gene 14137 | 1.199209 |
| 21961 | Tns1 | tensin 1 | 1.19977 |
| 209387 | Trim30d | tripartite motif-containing 30D | 1.213707 |
| 11670 | Aldh3a1 | aldehyde dehydrogenase family 3, subfamily A1 | 1.22835 |
| 209224 | Enox2 | ecto-NOX disulfide-thiol exchanger 2 | 1.231626 |
| 360013 | Myo18a | myosin XVIIIA | 1.234905 |
| 16985 | Lsp1 | lymphocyte specific 1 | 1.241758 |
| 232174 | Cyp26b1 | cytochrome P450, family 26, subfamily b, polypeptide 1 | 1.260063 |
| 69847 | Wnk4 | WNK lysine deficient protein kinase 4 | 1.269461 |
| 21929 | Tnfaip3 | tumor necrosis factor, alpha-induced protein 3 | 1.282639 |
| 237928 | Phospho1 | phosphatase, orphan 1 | 1.291273 |
| 625530 | Usp17le | ubiquitin specific peptidase 17-like E | 1.293731 |
| 73906 | 4833417C18Rik | RIKEN cDNA 4833417C18 gene | 1.306661 |
| 57277 | Slurp1 | secreted Ly6/Plaur domain containing 1 | 1.326249 |
| 235047 | Zfp809 | zinc finger protein 809 | 1.336169 |
| 21826 | Thbs2 | thrombospondin 2 | 1.340408 |
| 213649 | Arhgef19 | Rho guanine nucleotide exchange factor (GEF) 19 | 1.341616 |
| 24132 | Zfp53 | zinc finger protein 53 | 1.344426 |
| 56636 | Fgf21 | fibroblast growth factor 21 | 1.351222 |
| 667666 | Zfp600 | zinc finger protein 600 | 1.372351 |
| 105171 | Arrdc3 | arrestin domain containing 3 | 1.3739 |
| 319586 | Celf5 | CUGBP, Elav-like family member 5 | 1.378512 |
| 380969 | Nckap5l | NCK-associated protein 5-like | 1.382966 |
| 57349 | Ppbp | pro-platelet basic protein | 1.409154 |
| 14457 | Gas7 | growth arrest specific 7 | 1.412939 |
| 68777 | Tmem53 | transmembrane protein 53 | 1.421016 |
| 68275 | Rpa1 | replication protein A1 | 1.426296 |
| 116847 | Prelp | proline arginine-rich end leucine-rich repeat | 1.441889 |
| 100859931 | Gm20604 | predicted gene 20604 | 1.456858 |
| 12323 | Camk2b | calcium/calmodulin-dependent protein kinase II, beta | 1.477047 |
| 71898 | Apol9b | apolipoprotein L 9b | 1.496034 |
| 18828 | Plscr2 | phospholipid scramblase 2 | 1.549629 |
| 15953 | Ifi47 | interferon gamma inducible protein 47 | 1.588867 |
| 70717 | Medag | mesenteric estrogen dependent adipogenesis | 1.59357 |
| 18295 | Ogn | osteoglycin | 1.616565 |
| 276905 | Armc7 | armadillo repeat containing 7 | 1.649982 |
| 434232 | Iqck | IQ motif containing K | 1.676162 |
| 104027 | Synpo | synaptopodin | 1.713193 |
| 223672 | Apol9a | apolipoprotein L 9a | 1.719233 |
| 16776 | Lama5 | laminin, alpha 5 | 1.744161 |
| 20324 | Sdpr | serum deprivation response | 1.791109 |
| 72421 | Ttc30b | tetratricopeptide repeat domain 30B | 1.82397 |
| 319942 | A530016L24Rik | RIKEN cDNA A530016L24 gene | 1.824428 |
| 233552 | Gdpd5 | glycerophosphodiester phosphodiesterase domain containing 5 | 1.860438 |
| 56863 | Cldn9 | claudin 9 | 1.903187 |
| 16651 | Sspn | sarcospan | 1.931287 |
| 14788 | Gpr162 | G protein-coupled receptor 162 | 1.938599 |
| 14349 | Fv1 | Friend virus susceptibility 1 | 1.95633 |
| 71795 | Pitpnc1 | phosphatidylinositol transfer protein, cytoplasmic 1 | 1.978626 |
| 21897 | Tlr1 | toll-like receptor 1 | 1.986825 |
| 11496 | Adam22 | a disintegrin and metallopeptidase domain 22 | 2.088537 |
| 54615 | Npff | neuropeptide FF-amide peptide precursor | 2.101212 |
| 16414 | Itgb2 | integrin beta 2 | 2.244307 |
| 217082 | Hlf | hepatic leukemia factor | 2.332983 |
| 320916 | Wscd2 | WSC domain containing 2 | 2.468149 |
| 68024 | Hist1h2bc | histone cluster 1, H2bc | 2.510796 |
| 100043772 | Zfp850 | zinc finger protein 850 | 2.61891 |
| 19016 | Pparg | peroxisome proliferator activated receptor gamma | 2.681824 |
| 100038725 | Cep85l | centrosomal protein 85-like | 2.686501 |
| 100134861 | ChkbCpt1b | Chkb-Cpt1b readthrough transcript (NMD candidate) | 2.795859 |
| 18190 | Nrxn2 | neurexin II | 2.86507 |
| 226695 | Ifi205 | interferon activated gene 205 | 2.877317 |
| 14747 | Cmklr1 | chemokine-like receptor 1 | 2.88818 |
| 246727 | Oas3 | 2'-5' oligoadenylate synthetase 3 | 3 |
| 100040671 | Gm2897 | predicted gene 2897 | 3.145979 |
| 11484 | Aspa | aspartoacylase | 3.397216 |
| 229898 | Gbp5 | guanylate binding protein 5 | 3.402098 |
| 22228 | Ucp2 | uncoupling protein 2 (mitochondrial, proton carrier) | 3.421464 |
| 70045 | 2610528A11Rik | RIKEN cDNA 2610528A11 gene | 3.597346 |
| 18160 | Npr1 | natriuretic peptide receptor 1 | 3.972693 |
| 20148 | Dhrs3 | dehydrogenase/reductase (SDR family) member 3 | 4.822698 |
| 73656 | Ms4a6c | membrane-spanning 4-domains, subfamily A, member 6C | 5.044394 |
| 54525 | Syt7 | synaptotagmin VII | 9.764872 |
| 243538 | Ccdc37 | coiled-coil domain containing 37 | 9.813781 |
| 102238433 | Jmjd7-pla2g4b | predicted gene, 28042 | 9.876517 |
| 226075 | Glis3 | GLIS family zinc finger 3 | 9.994353 |
| 100504486 | Gdap10 | ganglioside-induced differentiation-associated-protein 10 | 10.03617 |
| 66821 | Bcs1l | BCS1-like (yeast) | 10.77314 |
| 17146 | Mageb2 | melanoma antigen, family B, 2 | 11.03617 |
| 100039192 | Tmem254c | transmembrane protein 254c | 11.68825 |
| NM_001305668 | - | - | 11.78545 |
| NM_001305803 | - | - | 12.14211 |
| NR_131218 | - | - | 12.31911 |
| 319172 | Hist1h2ab | histone cluster 1, H2ab | 12.50432 |
| 100039150 | Mup15 | major urinary protein 15 | 12.94946 |
| 21822 | Tgtp1 | T cell specific GTPase 1 | 13.25562 |
| 319170 | Hist1h2an | histone cluster 1, H2an | 13.42522 |

**Supplementary Table S4** The list of 41 DEGs common to the three pairwise treatment conditions in cultured HT22 cells.

| **Gene ID** | **Symbol** | **Description** |
| --- | --- | --- |
| 11670 | Aldh3a1 | aldehyde dehydrogenase family 3, subfamily A1 |
| 11677 | Akr1b3 | aldo-keto reductase family 1, member B3 (aldose reductase) |
| 16589 | Uhmk1 | U2AF homology motif (UHM) kinase 1 |
| 17101 | Lyst | lysosomal trafficking regulator |
| 18811 | Prl2c2 | prolactin family 2, subfamily c, member 2 |
| 19714 | Rev3l | REV3-like, catalytic subunit of DNA polymerase zeta RAD54 like (S. cerevisiae) |
| 20296 | Ccl2 | chemokine (C-C motif) ligand 2 |
| 21822 | Tgtp1 | T cell specific GTPase 1 |
| 21825 | Thbs1 | thrombospondin 1 |
| 59090 | Midn | midnolin |
| 66573 | Dzip1 | DAZ interacting protein 1 |
| 69576 | Smco1 | single-pass membrane protein with coiled-coil domains 1 |
| 70005 | Znf41-ps | ZNF41, pseudogene |
| 77057 | Ston1 | stonin 1 |
| 94352 | Loxl2 | lysyl oxidase-like 2 |
| 104027 | Synpo | synaptopodin |
| 109181 | Trip11 | thyroid hormone receptor interactor 11 |
| 208501 | 1810043H04Rik | RIKEN cDNA 1810043H04 gene |
| 211429 | Pla2g4b | phospholipase A2, group IVB (cytosolic) |
| 223922 | Atf7 | activating transcription factor 7 |
| 224836 | Usp49 | ubiquitin specific peptidase 49 |
| 226075 | Glis3 | GLIS family zinc finger 3 |
| 226251 | Ablim1 | actin-binding LIM protein 1 |
| 230895 | Vps13d | vacuolar protein sorting 13 D (yeast) |
| 240476 | Zfp407 | zinc finger protein 407 |
| 246727 | Oas3 | 2'-5' oligoadenylate synthetase 3 |
| 319586 | Celf5 | CUGBP, Elav-like family member 5 |
| 320871 | B230206H07Rik | RIKEN cDNA B230206H07 gene |
| 333789 | N4bp2 | NEDD4 binding protein 2 |
| 382090 | Cep162 | centrosomal protein 162 |
| 100039054 | Mup12 | major urinary protein 12 |
| 100039890 | Gm15093 | predicted gene 15093 |
| 100039934 | Gm15085 | predicted gene 15085 |
| 100041515 | Gm3383 | predicted gene 3383 |
| 100042149 | Gm3696 | predicted gene 3696 |
| 100859931 | Gm20604 | predicted gene 20604 |
| 102238433 | Jmjd7-pla2g4b | predicted gene, 28042 |
| NM_001305675 | - | - |
| NM_001305844 | - | - |
| NR_131218 | - | - |
| NR_131225 | - | - |

**Supplementary Table S5** Gene ontology analysis of DEGs among treatment conditions in HT22 cells. The top canonical pathways, diseases/disorders, and biological functions significantly associated with DEGs were predicted using IPA software. The Fisher’s exact test was used to calculate P-values that reflect the probability that the DEGs are not associated with the described pathway, disorder, or function. *P* < 0.05 is considered significant. A range of P-values are shown for the various subcategories of diseases/disorders or biological functions under a general category, such as cancer or lipid metabolism.

| **Categories** | **P-value** | **# DEGs** |
| --- | --- | --- |
| **Control_VS-5mMGlu** | | |
| ***Canonical Pathways*** | | |
| Hepatic Fibrosis / Hepatic Stellate Cell Activation | 1.31E-04 | 9 |
| Atherosclerosis Signaling | 3.13E-04 | 7 |
| Hepatic Fibrosis Signaling Pathway | 9.21E-04 | 12 |
| Pulmonary Fibrosis Idiopathic Signaling Pathway | 1.47E-03 | 10 |
| Apelin Liver Signaling Pathway | 2.03E-03 | 3 |
| ***Diseases/Disorders*** | | |
| Cancer | 1.72E-03 - 1.72E-08 | 215 |
| Organismal Injury and Abnormalities | 1.72E-03 - 1.72E-08 | 218 |
| Neurological Disease | 1.39E-03 - 2.27E-08 | 143 |
| Gastrointestinal Disease | 1.61E-03 - 2.69E-08 | 194 |
| Dermatological Diseases and Conditions | 1.39E-03 - 3.07E-08 | 166 |
| ***Biological Functions*** | | |
| Cardiovascular System Development and Function | 1.65E-03 - 3.67E-06 | 51 |
| Tissue Development | 1.69E-03 - 3.89E-06 | 77 |
| Skeletal and Muscular System Development and Function | 1.56E-03 - 3.92E-06 | 31 |
| Connective Tissue Development and Function | 1.39E-03 - 1.28E-05 | 44 |
| Organismal Development | 1.69E-03 - 1.61E-05 | 95 |
| **5mMGlu-VS-AE-Et50** | | |
| ***Canonical Pathways*** | | |
| Role of OCT4 in Mammalian Embryonic Stem Cell Pluripotency | 1.79E-04 | 5 |
| CSDE1 Signaling Pathway | 3.89E-03 | 4 |
| p53 Signaling | 5.46E-03 | 5 |
| DNA Double-Strand Break Repair by Homologous Recombination | 1.09E-02 | 2 |
| Bladder Cancer Signaling | 1.09E-02 | 5 |
| ***Diseases/Disorders*** | | |
| Cancer | 3.92E-03 - 5.27E-11 | 255 |
| Organismal Injury and Abnormalities | 3.92E-03 - 5.27E-11 | 259 |
| Gastrointestinal Disease | 3.22E-03 - 3.79E-09 | 234 |
| Reproductive System Disease | 3.71E-03 - 6.29E-09 | 191 |
| Neurological Disease | 3.16E-03 - 7.51E-09 | 181 |
| ***Biological Functions*** | | |
| Organ Morphology | 3.65E-03 - 1.47E-05 | 32 |
| Organismal Development | 3.86E-03 - 1.47E-05 | 98 |
| Renal and Urological System Development and Function | 3.51E-03 - 1.47E-05 | 18 |
| Cardiovascular System Development and Function | 3.65E-03 - 3.49E-05 | 45 |
| Visual System Development and Function | 3.64E-03 - 3.49E-05 | 6 |
| **5mMGlu-VS-SA-Et50** | | |
| ***Canonical Pathways*** | | |
| Tumor Microenvironment Pathway | 5.30E-07 | 15 |
| Hepatic Fibrosis / Hepatic Stellate Cell Activation | 1.47E-06 | 15 |
| GP6 Signaling Pathway | 2.07E-06 | 12 |
| Clathrin-mediated Endocytosis Signaling | 3.21E-05 | 13 |
| Wound Healing Signaling Pathway | 3.44E-05 | 15 |
| ***Diseases/Disorders*** | | |
| Cancer | 1.67E-06 - 4.51E-21 | 388 |
| Organismal Injury and Abnormalities | 1.67E-06 - 4.51E-21 | 395 |
| Endocrine System Disorders | 1.25E-06 - 1.59E-18 | 350 |
| Reproductive System Disease | 1.15E-06 - 1.61E-17 | 301 |
| Renal and Urological Disease | 1.52E-06 - 5.91E-16 | 149 |
| ***Biological Functions*** | | |
| Cardiovascular System Development and Function | 1.60E-06 - 8.12E-23 | 128 |
| Organismal Development | 1.60E-06 - 8.12E-23 | 190 |
| Tissue Development | 1.59E-06 - 4.45E-18 | 174 |
| Organ Morphology | 1.60E-06 - 5.91E-16 | 121 |
| Renal and Urological System Development and Function | 1.45E-06 - 5.91E-16 | 47 |

**Supplementary Table S6** Neurological diseases and functions significantly associated with DEGs among treatment conditions in HT22 cells. The list of DEGs from RNA-seq analysis were analyzed using IPA software to predict neurological diseases and functions associated with DEGs. P-values were calculated using Fisher's exact test. *P* < 0.05 is considered significant.

| **Name** | **P-value** | **Number of Genes** |
| --- | --- | --- |
| **Control_VS-5mMGlu** | | |
| **Neurological Disease** |  |  |
| Cerebral disorder | 2.06E-06 | 76 |
| Central nervous system cancer | 2.76E-06 | 137 |
| Brain glioma | 4.27E-06 | 133 |
| Glioma | 6.18E-06 | 136 |
| Cerebrovascular dysfunction | 4.73E-04 | 13 |
| **Nervous System Development and Function** |  |  |
| Sensory system development | 4.35E-04 | 18 |
| Activation of microglia | 4.88E-04 | 7 |
| Differentiation of synapse | 5.64E-04 | 2 |
| Morphogenesis of nervous tissue | 7.00E-04 | 21 |
| Neuritogenesis | 1.38E-03 | 20 |
| **5mMGlu-VS-AE-Et50** | | |
| **Neurological Disease** |  |  |
| Brain lesion | 7.51E-09 | 165 |
| Central nervous system cancer | 2.22E-08 | 165 |
| Cerebral disorder | 3.44E-08 | 92 |
| Early-onset Alzheimer disease | 1.26E-03 | 3 |
| Congenital neurological disorder | 2.59E-03 | 28 |
| **Nervous System Development and Function** |  |  |
| Morphogenesis of neurons | 6.65E-05 | 26 |
| Neuritogenesis | 1.40E-04 | 25 |
| Development of neurons | 4.52E-04 | 29 |
| Differentiation of neurons | 5.56E-04 | 17 |
| Development of central nervous system | 3.84E-03 | 22 |
| **5mMGlu-VS-SA-Et50** | | |
| **Neurological Disease** |  |  |
| Cerebral disorder | 4.47E-15 | 148 |
| Central nervous system cancer | 2.63E-12 | 249 |
| Brain glioma | 4.76E-11 | 239 |
| Brain astrocytoma | 1.04E-07 | 180 |
| Familial encephalopathy | 1.49E-06 | 78 |
| **Nervous System Development and Function** |  |  |
| Sensory system development | 7.24E-11 | 41 |
| Formation of eye | 9.63E-11 | 38 |
| Morphology of eye | 5.68E-09 | 28 |
| Development of neurons | 7.66E-08 | 50 |
| Abnormal morphology of retina | 6.06E-07 | 18 |

**Supplementary Table S7** Specific primers used for qRT-PCR.

| **Gene** | **Sequence** | **Product size (bp)** |
| --- | --- | --- |
| *Apoe* | Forward primer 5ʹ GTGCTGTTGGTCACATTGCT 3ʹ | 257 |
|  | Reverse primer 5ʹ AGCTGTTCCTCCAGCTCCTT 3ʹ |  |
| *Ptgs2* | Forward primer 5ʹ CAACTCCATCCTCCTGGAAC 3ʹ | 241 |
|  | Reverse primer 5ʹ TTCTGCAGCCATTTCCTTCT 3ʹ |  |
| *Rest* | Forward primer 5ʹ GACACATGCGGACTCATTCA 3ʹ | 122 |
|  | Reverse primer 5ʹ AAGAGGTTTAGGCCCGTTGT 3ʹ |  |
| *Zbed6* | Forward primer 5ʹ GCCTGCCAGATTTCAAGTTC 3ʹ | 238 |
|  | Reverse primer 5ʹ GCCAGTAAACCAAAGGGTCA 3ʹ |  |
| *Loxl2* | Forward primer 5ʹ GCCAACTTTGGAGAACAAGG 3ʹ | 215 |
|  | Reverse primer 5ʹ TTGTACATCCAGATGCGGTAG 3ʹ |  |
| *Ccl2* | Forward primer 5ʹ AGGTCCCTGTCATGCTTCTG 3ʹ | 249 |
|  | Reverse primer 5ʹ TCTGGACCCATTCCTTCTTG 3ʹ |  |
| *Synpo* | Forward primer 5ʹ GAAGAGGCCGATTGACAGAG 3ʹ | 189 |
|  | Reverse primer 5ʹ GCCTGTTGAAGAGCTGGAC 3ʹ |  |
| *Ablim1* | Forward primer 5ʹ AGGAGTATCCATGCCCAACA 3ʹ | 209 |
|  | Reverse primer 5ʹ TCTTCATGTCGTTGCGTCTC 3ʹ |  |
| *Glis3* | Forward primer 5ʹ TCCATCTCCTCACCACATCA 3ʹ | 271 |
|  | Reverse primer 5ʹ CAGGCAGTCCTCAAAGGAAG 3ʹ |  |
| *β-actin* | Forward primer 5ʹ GGCTGTATTCCCCTCCATCG 3ʹ | 154 |
|  | Reverse primer 5ʹ CCAGTTGGTAACAATGCCATGT 3ʹ |  |
